# Predicting and finding geroprotective compounds through modulating conserved longevity–associated aging modules

**DOI:** 10.1126/sciadv.aee3657

**Published:** 2026-09-30

**Authors:** Weihan Huai, Li-Fang Ng, Jasinda H. Lee, Qingyun Wang, Kamil Pabis, Zhuangli Yee, Shivaanisha Raventhiran, Shou-Ping Guan, Shermaine Thein, Stephen Dinesh Raj, Jerald Tan, Angelica Faye Ga, Sharah Mae Capinpin, Diogo Barardo, Qing Zhang, Zongmin Liu, Esther Wong, Brian Keith Kennedy, Jan Gruber

**Affiliations:** ^1^ Department of Biochemistry, Yong Loo Lin School of Medicine, National University of Singapore, Singapore 117596, Singapore.; ^2^ Healthy Longevity Translational Research Programme, Yong Loo Lin School of Medicine, National University of Singapore, Singapore 117456, Singapore.; ^3^ Centre for Healthy Longevity, National University Health System, Singapore 159964, Singapore.; ^4^ Science Divisions, Yale, NUS College, Singapore 138527, Singapore.; ^5^ Department of Physiology, Yong Loo Lin School of Medicine, National University of Singapore, Singapore 117593, Singapore.

## Abstract

Currently, no generalizable tools exist for predicting the impact of interventions on aging using gene expression changes. Here, we build a model for predicting potential geroprotectors by deconstructing the aging transcriptome into coexpression modules and identifying those modulated by life span–extending interventions in *Caenorhabditis elegans* (*C. elegans*). Many of these modules exhibited adaptive changes during aging that are linked to longevity. Using these longevity-associated modules, we predicted and validated 10 compounds (7 previously unreported) that significantly extended life span in *C. elegans*. RNA sequencing analysis of two top candidates also showed targeted modulation of our longevity-associated modules. We identified conservation of several modules in mice that were also correlated with frailty. Together, our findings demonstrate that beneficial transcriptomic adaptations occur during aging and that targeting longevity-associated modules offers a framework for geroprotector discovery.

## INTRODUCTION

Aging is characterized by continuous deterioration of resilience and an increase in risk of diseases, leading to a gradual decline in function and, ultimately, death. For years, extensive efforts have been made to identify the root causes or hallmarks of aging, leading to the development of various therapeutic strategies—such as small molecules, epigenetic reprogramming, and stem cell therapy—targeting these mechanisms (*1*–*4*). Among these strategies, small molecules—often termed geroprotectors—have gained particular attention. Notably, clinical trials on metformin and rapamycin, two of the most well-characterized geroprotectors, are underway to test their ability to prevent disease and slow or reverse age-associated biomarkers (*5*, *6*).

To date, invertebrate-based screens have identified hundreds of life span–extending compounds (*7*). However, relatively few have been validated across multiple species, and most of the geroprotectors belong to extensively studied classes like mechanistic target of rapamycin (mTOR) inhibitors, AMPK activators, or histone deacetylase (HDAC) inhibitors. Because these pathways regulate broad cellular processes, direct pharmacological modulation of their core components may produce pleiotropic effects. This reliance on a limited set of well-studied targets and drug classes underscores the need for unbiased tools that can prioritize diverse candidate geroprotectors.

Currently, several strategies have been developed to identify small-molecule geroprotectors, ranging from unbiased screening to transcriptomic-based drug prediction. The latter, which infers the geroprotective effects of drugs based on drug-induced transcriptomic changes, is an emerging method and has gained traction thanks to availability of large-scale transcriptomic resources like the Aging Atlas and Connectivity Map (CMap) (*8*, *9*). Several methodologies have been developed within this framework. One widely used strategy is the “reversal approach,” which identifies drugs that counteract aging-associated gene expression changes. This approach has led to discoveries such as oridonin, an AKT inhibitor extending life span in yeast and mice (*10*). Another application of reversal approach involves using aging clocks to identify drugs whose transcriptomic profiles correspond to a lower biological age, which helped find Hsp90 inhibitors as geroprotector candidates (*11*). However, the reversal approach assumes that all age-related changes are detrimental despite evidence that some may be adaptive (*12*). Another strategy, the “mimic approach,” matches drug-induced transcriptomic profiles to those of known antiaging interventions. This method has been applied to identify compounds like allantoin and rilmenidine, which extend life span in *Caenorhabditis elegans* (*13*, *14*). However, this method tends to rediscover drugs similar to known interventions, limiting its capacity to reveal novel mechanisms.

Here, we used *C. elegans* and mice to explore transcriptomic strategies for geroprotector discovery. Applying transcriptomic clocks and coexpression network analysis, we found that many aging-associated changes in *C. elegans* are adaptive—Some transcriptomic changes that resemble aging may contribute to longevity. We identified conserved coexpression modules associated with life span: “aging modules” enriched in translation and mitochondrial genes and “longevity modules” enriched in stress response genes, including *daf-16* targets. Several of these longevity-associated modules are preserved in specific mouse tissues and are associated with mouse health span. Last, we conducted a small-scale screen and identify seven novel geroprotectors using the module gene expressions as our transcriptomic signatures. Our findings demonstrate that expression profiles of conserved aging-related processes can serve as predictive signatures for finding effective geroprotective compounds.

## RESULTS

### Transcriptomic clocks show limited predictive power for pharmacological interventions

To test whether longevity interventions can be identified through the reversal approach, we performed RNA sequencing (RNA-seq) to *C. elegans* from five ages (days 6, 8, 11, 14, and 18, six replicates each), along with 30 wild-type and 20 short-lived *daf-16*(*mu86*) mutant samples collected at day 6 from hatching. Following our approach to construct principal components (PCs) analysis (PCA)–based omics clocks in *C. elegans* and humans (*15*, *16*), we split the wild-type data into train and test data and then trained a transcriptomic clock using elastic net regression on the top 20 PCs of the train data. PCA on wild-type train data revealed that PC1 is strongly correlated with age (Spearman ρ = 0.88), while other PCs had minimal association (Fig. 1, A and B, fig. S1B). As expected, PC1 received the highest weight, and the model accurately predicted age in held-out wild-type test data (table S1 and fig. S1C). The clock also captured the accelerated aging in *daf-16*(*mu86*) mutants (ΔAge = +0.52, *P* = 2.6 × 10^−6^) (Fig. 1C and fig. S1D) and was able to detect delayed aging in long-lived *eat-2* mutants using the data from our previous study (*17*) (ΔAge = −4.04, *P* = 7 × 10^−5^) (Fig. 1, C and D).

**Fig. 1.**
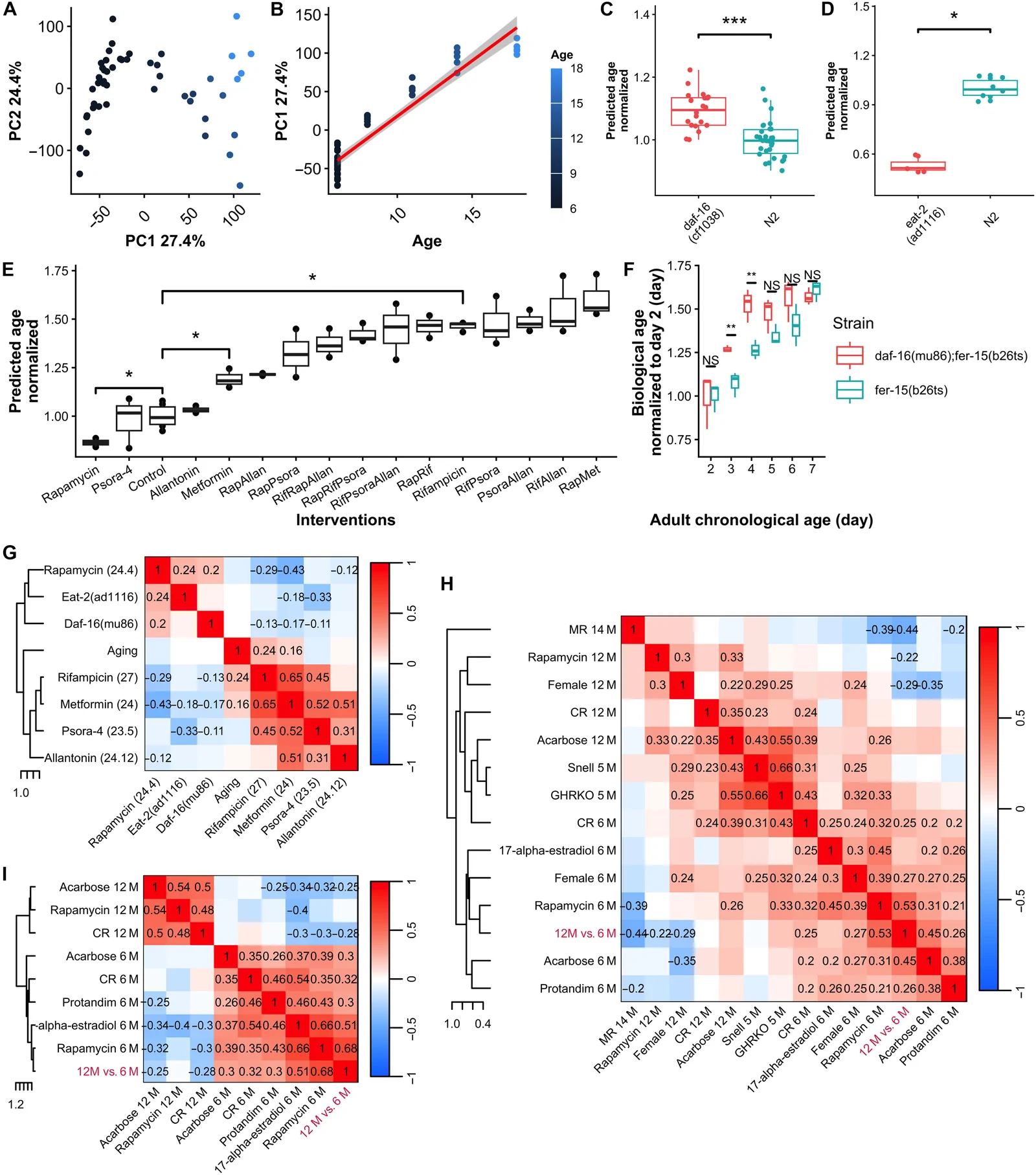
Model aging transcriptome using PCA clock and application of PCA clock to explain interventions. (**A**) PCA of aging wild-type samples in the train data from day 6 to day 18. (**B**) Association of PC1 with ages. (**C**) Biological age prediction of short-lived *daf-16*(*mu86*) mutants in this study. (**D**) Biological age prediction of *eat-2*(*ad1116*) mutant comparing with wild-type in GSE108263. (**E**) Biological age prediction of drug interventions comparing with control in GSE108263. (**F**) Predicted ages of germline-free *fer-15*(*b26ts*) control and *fer-15*(*b26ts*);*daf-16*(*mu86*) mutants from adult day 2 to adult day 7 in GSE122892. (**G**) Correlation of Z statistics of genes differentially expressed for individual drug intervention contrast, aging, and mutant in GSE108263 and this dataset. Only correlations with absolute value >0.15 are shown explicitly. Correlation matrix was clustered using the average hierarchical clustering. (**H**) Spearman correlation of Z statistics of DEGs for 12 M versus 6 M male for individual intervention contrast and gender contrast in male mice in GSE131901. (**I**) Spearman correlation of Z statistics of DEGs for 12 M versus 6 M female for individual intervention contrast in female mice in GSE131901. NS. not significant; *P <= 0.05; **P <= 0.01; ***P <= 0.001.

However, when we applied our transcriptomic clock to the transcriptomic data of worms treated with geroprotective drugs performed in our previous study (*17*), we found that most treatments resulted in transcriptomic ages exceeding chronological age, except for rapamycin, which reduced transcriptomic age (Fig. 1E). To test whether this limitation was specific to our transcriptomic clock, we applied BitAge (*18*), a previously published *C. elegans* transcriptomic clock, to the same dataset. Like the PCA clock, we were unable to observe expected younger transcriptomic age of drug-treated worms using BitAge (fig. S1E). These findings show that short-term pharmacological interventions may not reverse age-associated transcriptomic changes and suggest that the assumption from the reversal approach that reversing aging-dependent changes are beneficial is often not true.

We hypothesized that part of the transcriptomic changes during aging were driven by adaptive responses to aging, some of which might be activated by drug interventions. To test this explanation, we compared DE Z-statistics between drug-treated and aging worms. Metformin and rifampicin, both of which increased transcriptomic age, showed positive correlations with aging signatures (ρ = 0.16 to 0.22). Similar positive correlations are also observed in drug combinations, suggesting that these drugs mimic a part of age-associated gene expression (Fig. 1E and fig. S1F).

To further show that a part of these age-associated changes can be protective, we further selected *daf-16* target genes previously determined by Tepper *et al.* (*19*) that were activated during aging. We found a significant overlap between genes down-regulated during aging and class II *daf-16* targets (up-regulated upon *daf-16* suppression in *daf-2* mutant background) (fig. S1G), indicating that a part of the transcriptomic changes are contributed by *daf-16* activation and support the idea that a portion of age-associated transcriptomic changes may reflect adaptive responses. We found that the DE Z-statistics of rifampicin and metformin showed significant positive correlation with aging signatures (ρ = 0.15 to 0.18) on these *daf-16* target genes (fig. S1H). This indicates that metformin and rifampicin may activate protective, *daf-16*–dependent programs that partially overlap with aging-like signatures. *daf-16*(*mu86*) mutants, while exhibiting opposite Z-statistic correlation with aging on these genes, were predicted to be transcriptomically older, suggesting the presence of *daf-16*–independent pathways that drive the biologically older *daf-16*(*mu86*) mutants.

Supporting our observation, *daf-16*(*mu86*) mutants plateaued in transcriptomic age after day 7 in an independent dataset (*20*), while controls showed continuous increase in transcriptomic age (Fig. 1F), implying that a part of transcriptomic age changes during aging are adaptive and driven by *daf-16* activation. Consistently, long-lived *daf-2*(*e1370*) mutants, which have sustained *daf-16* activation, were predicted across four independent datasets to have either comparable or significantly older transcriptomic ages than N2 controls by both our PCA-based aging clock and BitAge clock (fig. S1I). These findings further support the notion that early or sustained activation of adaptive responses could result in older transcriptomic ages.

To assess whether this phenomenon is preserved across species, we reanalyzed the mouse liver transcriptomic data from a previous study testing genetic or pharmaceutical interventions that extended life span (*14*). We selected differentially expressed genes (DEGs) between 12 and 6 months and compared their DE Z-statistics upon intervention at different time points. Similarly, early interventions (at 6 months) induced aging-like expression (e.g., rapamycin: ρ = 0.36 to 0.54), but these effects diminished or reversed by 12 months (particularly in females), mirroring our findings in worms (Fig. 1, H and I).

Together, these findings show that a subset of gene expression changes during aging are adaptive processes that were also induced by early longevity interventions. As a result, the effectiveness of using the reversal approach to find novel geroprotectors is limited, particularly since most drug-induced transcriptomic profiles are collected during the early phase of the drug treatment. Our results suggest a potential way to predict geroprotectors by delineating longevity-associated adaptive and nonadaptive changes from the aging transcriptome. To distinguish these changes, we propose modeling aging as a network of coexpressed gene modules, enabling functional classification and improved prediction of geroprotective interventions.

### Constructing aging/*daf-16*–associated homeostatic network using WGCNA

To delineate adaptive and non-adaptive changes, we applied weighted gene coexpression network analysis (WGCNA) to aging *C. elegans* wild-type and *daf-16*(*mu86*) samples (*21*, *22*). WGCNA groups coexpressed genes into modules, capturing coexpressed regulatory patterns that could not be detected by matrix factorization methods such as PCA.

Using an optimized tree-cutting strategy, we identified 38 main modules and 62 submodules (Fig. 2A and fig. S2A). We used module eigengenes, which is defined as the first PC of each module, as the representative of the module’s collective expression level in each sample. We correlated module eigengenes with age and *daf-16* status. Of 62 submodules, 39 were age associated, 26 responded to *daf-16*, and 19 responded to both—revealing overlapping and distinct transcriptomic effects.

**Fig. 2.**
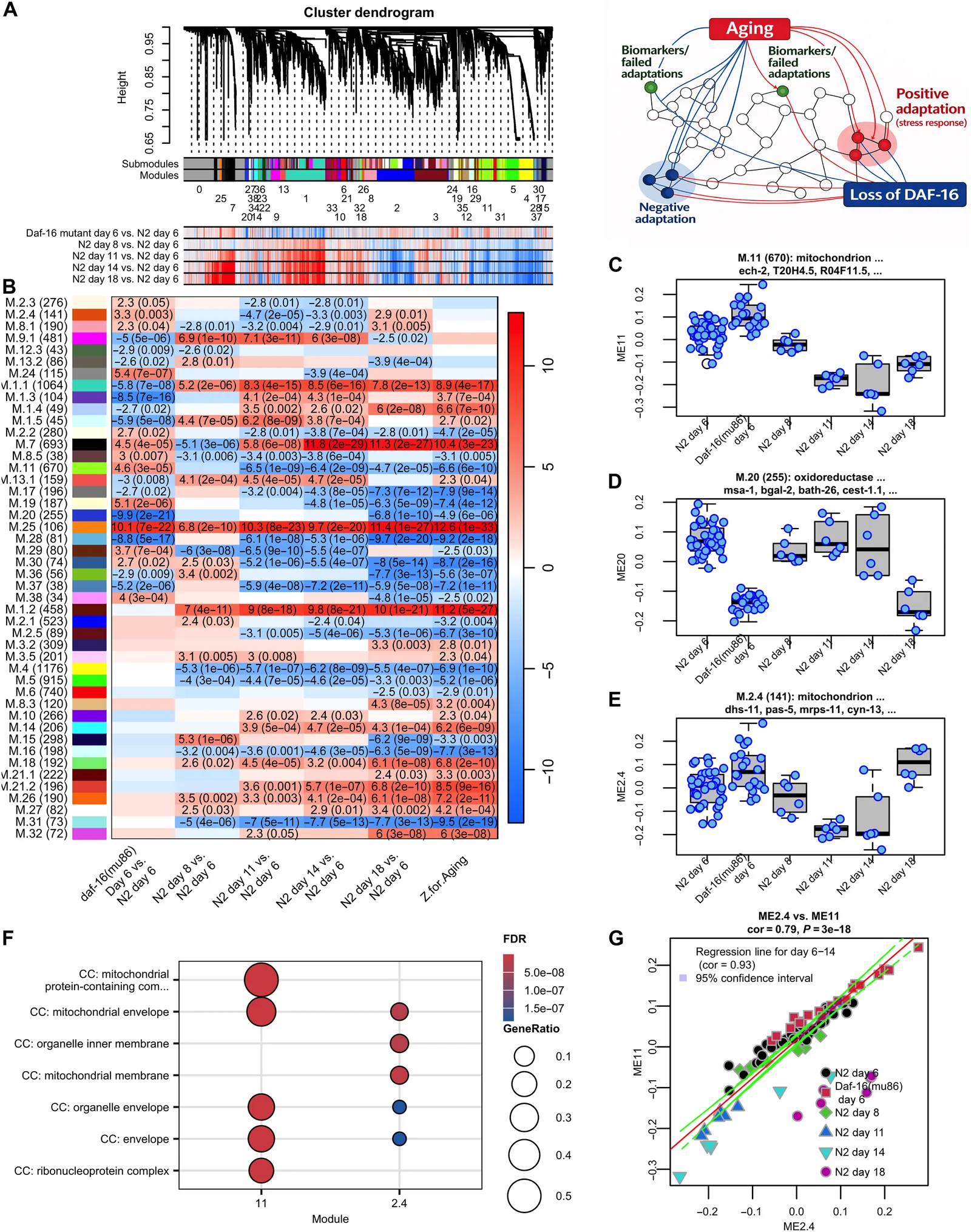
Coexpression network analysis on aging and *daf-16*(*mu86*) mutant worms. (**A**) (Left) Dendrogram with module colors, labels, and heatmap of individual gene association with age contrasts or *daf-16*(mu86) and (right) schematic of module association with loss of *daf-16* and aging and their possible role. Nodes are coexpression modules, and edges are associations. The color of the edges represents positive (red) or negative (blue) associations. Created in BioRender. Huai, W. (2026) https://BioRender.com/3x07ep7. (**B**) Association module eigengenes with selected submodules with contrast of ages, mutants, and overall age. The labels of each row indicate the numerical module numbers and the number of genes in the given module. Each column represents a binarized contrast of wild-type ages versus day 6 or *daf-16*(mu86) versus wild-type or overall association with ages. The numbers in the heatmap give the association significance Z statistics with the FDR estimates, which is only shown for those with FDR < 0.05. (**C** to **E**) Expression of M11, M20, and M2.4 across ages and strains. For each plot, the title shows the enrichment label for the module (top enrichment terms) and the top hub genes (ordered by decreasing kME) in each module. (**F**) Enrichment dotplot of module M11 and M2.4. (**G**) Scatterplot of module eigengene M11 with M2.4.

To compare modules with PC1 in the aging clock, we mapped the top and bottom 1000 weighted genes in PC1 from our PCA clock to the WGCNA modules. As expected, PC1 genes from our aging clock were strongly enriched in modules associated with age, including M1.2, M4, and M7 (e.g., *P* = 0 to 1 × 10^−120^) (Fig. 2B and fig. S2B). Even modules weakly correlated with age, like M11, showed strong enrichment, suggesting PC1 captures age-related modules and mixes them together (fig. S2C).

We hypothesized that modules showing opposite regulation between aging and *daf-16* loss may capture adaptive responses, while concordant changes represent nonadaptive changes, which might include aging biomarkers or causative processes (Fig. 2A). Of the 19 modules associated with both aging and *daf-16* mutation, 12 showed opposite regulation—including M11, enriched in mitochondrial genes (Fig. 2, B and C). The remaining seven modules, such as M20, changed in the same direction. M20, enriched in oxidoreductases and genes with *daf-16* transcription factor–binding motifs, was down-regulated during both aging and *daf-16* loss (Fig. 2, B and D) and significantly enriched in class I *daf-16* targets (*P* = 2 × 10^−61^) (*19*), supporting its role in life span regulation (fig. S2D). Coexpression among *daf-16*–associated modules increased after day 8, suggesting coordinated activation postreproduction (fig. S2H).

Notably, not all *daf-16*–associated modules overlapped with known *daf-16* targets. For example, M2.4 showed significant association with *daf-16* but minimal enrichment for *daf-16* target, indicating that their regulation may reflect broader systemic shifts. (number of overlap = 9, *P* = 1 for class II) (table S4). To better characterize the transcriptional impact of *daf-16* mutation, we performed DE analysis with 36 wild-type and 20 *daf-16*(*mu86*) mutants to generate a high-resolution list of genes differentially expressed upon *daf-16* mutation (table S2). When compared with previously defined *daf-16* targets and chromatin immunoprecipitation sequencing (ChIP-seq) results, we found that approximately 4000 DEGs (FDR ≤ 0.05) were unique to our dataset, suggesting broader transcriptomic changes beyond direct *daf-16* inactivation (fig. S2E).

Notably, modules M7 and M4, which were modules with the highest weights on our PCA-based aging clock (fig. S2C), were regulated in the same direction by both *daf-16* mutation and aging despite lacking direct *daf-16* targets. This suggests that these modules may contribute to the elevated transcriptomic ages observed in *daf-16*(*mu86*), even in the context of suppressed adaptive responses. Module M7 was enriched in genes anatomic associated with gonad and sperms (table S3), indicating that dysregulation of spermatogenesis may serve as a biomarker or potential driving factor of aging in *C. elegans*. Such dysregulation could partially account for the increased transcriptomic ages of *daf-16*(*mu86*) worms.

Mapping GenAge (*23*) genes revealed that module M27 was enriched in antilongevity genes—genes whose mutation and/or knock-down extend life span—suggesting that global down-regulation of M27 may contribute to longevity (fig. S2F). We observed a shift in expression of modules around day 18, including mitochondrial modules M11 and M2.4 and *daf-16* modules M20 and M1.3 (Fig. 2, C to E, and fig. S2G). The expression of M11 and M2.4, which shared mitochondrial enrichment (Fig. 2, F and G), decreases between day 6 and day 14 but then increased significantly by day 18. Moreover, their module expression correlation dropped from 0.93 (pre-day 18) to 0.79, suggesting that mitochondrial genes are less correlated at day 18 (Fig. 2H).

Together, these findings suggest that aging involves both nonadaptive changes and adaptive transcriptomic responses. Transcriptional drift and coherence loss late in life may mark dysregulation of physiological control. To explore how interventions modulate our modules and why transcriptomic clocks fail, we examined how they are perturbed upon drug interventions.

### Inferring module association with longevity

To evaluate the stability of aging/*daf-16* coexpression modules under drug treatment, we applied WGCNA to our intervention dataset (GSE108263) and assessed the preservation between aging/*daf-16* submodules and drug modules. Using the permutation-derived Zsummary statistic and previously established thresholds (*24*, *21*), we found that 39 of 62 aging/*daf-16* submodules are strongly preserved (Zsummary ≥8), while 11 showed moderate preservation (Zsummary = 4 to 8), supporting the robustness of aging/*daf-16* modules in capturing intervention-driven transcriptomic changes. Similarly, 18 of 23 drug submodules showed at least moderate preservation (Fig. 3A and fig. S3C).

**Fig. 3.**
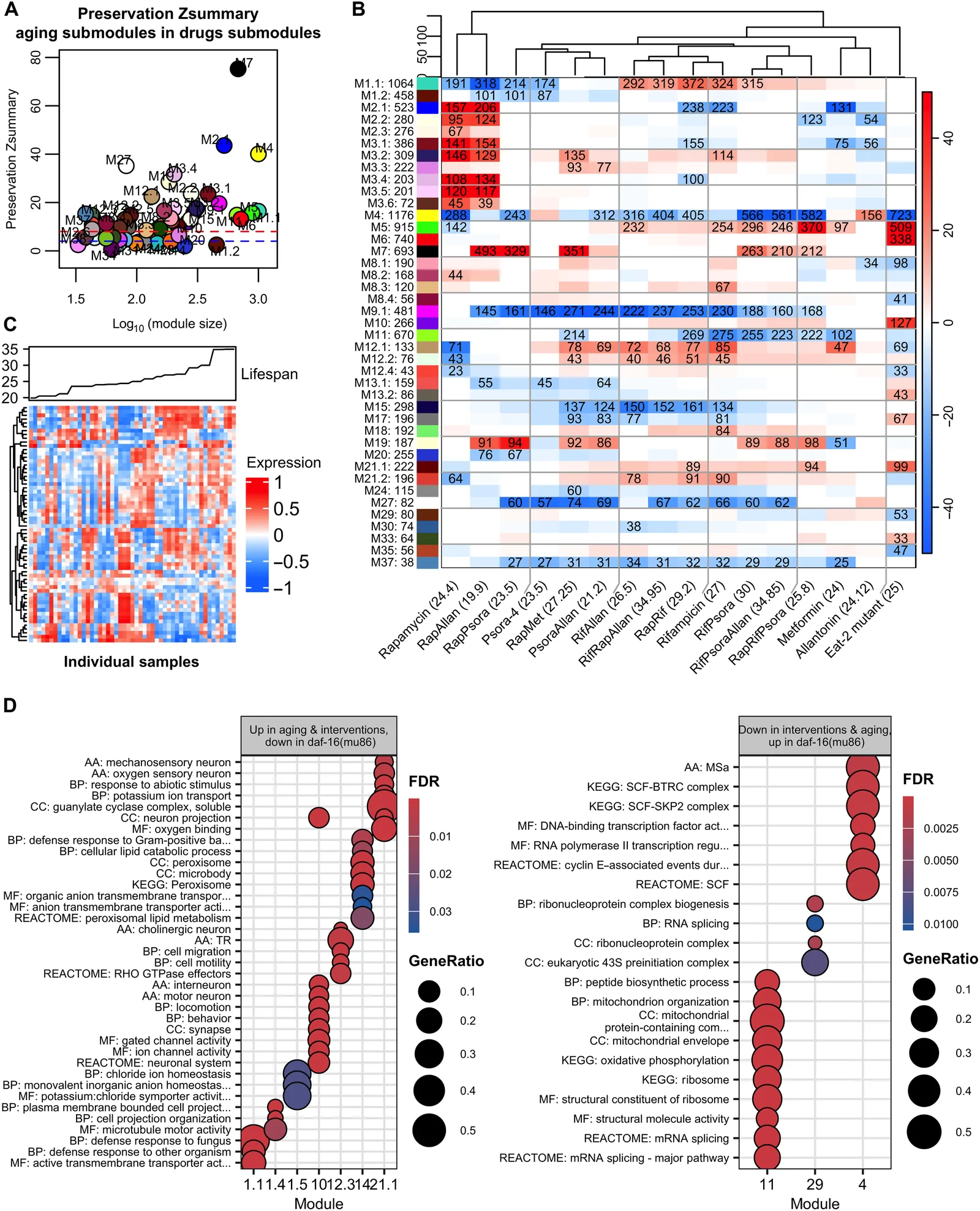
Perturbation of modules by drug interventions. (**A**) Preservation Zsummary statistics of aging/*daf-16* submodules in submodules derived from drug intervention dataset (GSE108263). (**B**) Enrichment of modules in genes DE at *P* < 0.01 in drug-treated worms or *eat-2* mutants versus controls. The method is first described in Wang et al. (*26*). Each row represents an aging/*daf-16* module with number of genes in the module and module color. Each column represents a treatment or a strain with the expected median life span extracted from original paper. Color in the heatmap indicates signed enrichment significance (−log_10_ of the Fisher’s exact test *P* value, negative and positive for down- and up-regulated genes, respectively). For each cell, the direction with lower *P* value (higher −log_10_ *P* value) is shown. Only those modules with least one *P* value <10^−10^ in all conditions are shown. Modules and perturbations are clustered using average linkage hierarchical clustering based on correlation of the signed significance. For each cell, the number of up- or down-regulated genes are shown for cells with *P* value <10^−10^. (**C**) Heatmap of GSVA scores of submodules associated with life span. *X* axis represents individual samples. The top panel showed the reported median life span of each condition extracted from the original paper. (**D**) Dot plot showing significantly enriched terms, ranked by adjusted *P* value, for (left) modules that are up-regulated both in interventions and during aging but down-regulated in *daf-16*(*mu86*) mutants, and (right) modules that are down-regulated both in interventions and during aging, while up-regulated in *daf-16*(*mu86*) mutants. For clarity, only the top two pathways from each gene set category (for example, GO and BP) are shown for each module. The complete enrichment table for all module is provided in table S2.

DE analysis confirmed that all interventions significantly enriched genes in at least one aging/*daf-16* submodule (*P* < 1 × 10^−10^) (Fig. 3B and table S3), suggesting selective module perturbation. The dietary restriction (DR) mimic *eat-2* clustered separately, while rifampicin grouped with life span–extending drug combinations and rapamycin with the life span–shortening rapamycin/allantoin (RapAllan) combination. Notably, down-regulated genes from rifampicin and combination treatments were enriched in modules M4 and M11—both negatively weighted in PC1—explaining the PCA clock’s prediction of older transcriptomic age.

We defined aging modules as those contributing to age-related decline and longevity modules as those enriched in processes beneficial for longevity. We hypothesized that these modules can be inferred through their behaviors under genetic or pharmacological interventions. To associate modules with longevity, we used the Gene Set Variation Analysis (GSVA) algorithm (*25*) quantify module activity and correlated with reported median life span (Fig. 3C).

Twenty of 62 submodules showed significant associations (FDR < 0.05), including 12 positive and 8 negatives (fig. S3D). We categorized modules as potential aging modules if they were up-regulated in the *daf-16*(*mu86*) mutants and negatively associated with median life span in the intervention dataset, and as longevity modules if they exhibited the reverse pattern. Among the 10 potential longevity modules, 8 showed increased expression with age, suggesting their adaptive activation during aging (table S4). These modules are enriched in gene anatomically associated with neurons and intestine, potentially contributing to the maintaining of functionality of these tissue, and genes associated with pathways such as lipid metabolisms and defense responses (Fig. 3D). We also found three potential aging modules that are down-regulated during aging, which were enriched in genes involved in essential metabolic turnover such as mitochondria, translation complexes, and proteasome-related SCF complexes, suggesting that the metabolic turnover drives aging in *C. elegans*, and its reduction during aging is potentially a part of the adaptive process (Fig. 3D). To minimize bias from individual compounds such as rifampicin, we evaluated conserved aging and longevity modules across public datasets to identify robust transcriptomic signatures of antiaging interventions.

### Meta-analysis to identify conserved longevity-associated modules

To identify conserved longevity-associated modules responsive to longevity interventions, we performed a meta-analysis of 25 RNA-seq datasets capturing diverse genetic and pharmacological perturbations. Only samples between day 3 and day 6 (after development and before aging) were used for consistency. Module activity was scored using GSVA, and associations with median life span were assessed via meta-analysis, stratified by gene targets and intervention types (Fig. 4A and table S5). To compare module association with the magnitude of life span extension, we also applied a mixed-effects meta-analysis using whether interventions belonging to the same study and the same control group as a random effect (Fig. 4B and table S5).

**Fig. 4.**
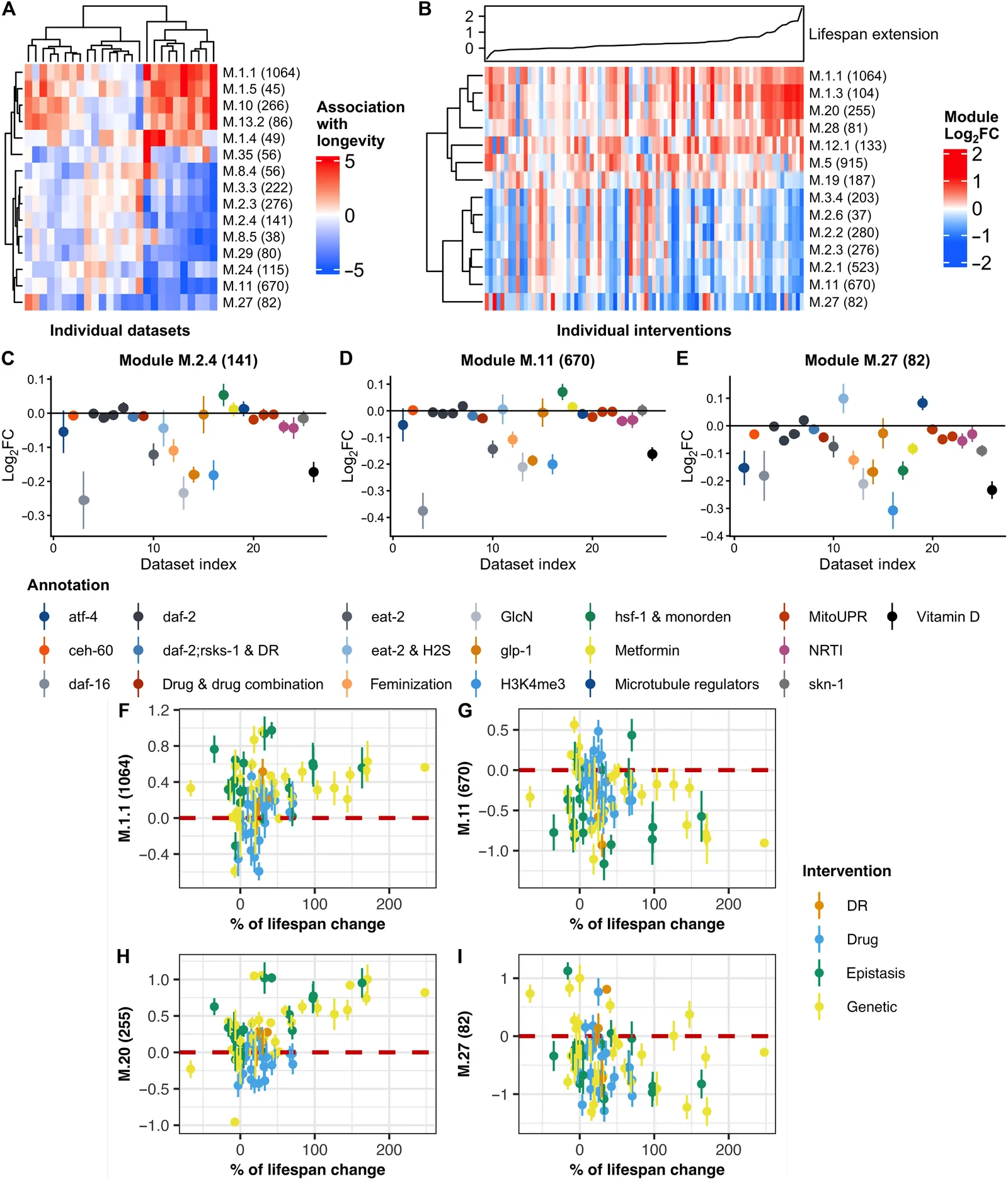
Meta-analysis on module association with life span across datasets. (**A**) Heatmap of association of longevity-associated modules with reported median life span across datasets. Colors indicate *z* scores of the *P* values from the meta-analysis. (**B**) Log_2_ fold change of module expression for each intervention versus its wild-type control with dataset numbers. Interventions were ranked on the basis of their effect on life span. (**C**) Association of modules M2.4 GSVA scores with reported median life span across datasets. Datasets are annotated on the basis of the major focus of study or epistasis of the dataset. (**D**) Association of module M11 GSVA scores with reported median life span across datasets. (**E**) Association of module M2.4 GSVA scores with reported median life span across datasets. (**F** to **I**) Association of module (F). M20, (G). M1.1, (H). M11, and (I). M27 expression change with extent of life span extension. All specified modules are significantly associated with life-span extension with FDR ≤ 0.05. Each condition versus control was colored on the basis of the type of the intervention. Genetic intervention includes mutation, knock-down, knockout, and overexpression; DR includes *eat-2* mutants or DR; epistasis includes combination of genetic or drug interventions where the life span extension is compromised comparing with single genetic or drug intervention.

Using the random-effect model, we identified 15 modules significantly associated with longevity (FDR ≤ 0.1), including 6 with stronger significance (FDR ≤ 0.05) (Fig. 4A). Ten showed negative associations, meaning that decreased expression of these modules was associated with increased life span (aging modules), notably mitochondrial modules M2.4 and M11 (Fig. 4, C and D), and the ribosomal module M27 (Fig. 4E). M27 had the strongest association and was suppressed in long-lived *nuo-6* and *atfs-1* gain-of-function mutants and activated in *atfs-1* loss-of-function animals. ATFS-1 loss reversed M27 suppression in *nuo-6*, consistent with its role in life span extension (fig. S4A) (*27*).

Using the mixed-effect model, 14 submodules were significantly associated with extent of life span extension (FDR ≤ 0.05) (Fig. 4B). Notably, M20 and M1.3—enriched for *daf-16* targets—showed the strongest positive associations (FDR = 7.4 × 10^−5^), suggesting that our method can capture established prolongevity biological pathways (Fig. 4F and fig. S4B). Modules M11, M1.1, and M27 were identified as aging modules, further supporting their relevance (Fig. 4, G to I). M11 and M27 expression positively correlated with life span extension under DR, but negatively under drug/genetic interventions, suggesting context-specific regulatory dynamics.

In summary, this meta-analysis identifies conserved mitochondrial, ribosomal, and proteostasis-related modules as longevity-associated modules, suggesting that a prolongevity intervention is likely to be observed to perturb the expression of genes within these modules. These modules provide insight into aging mechanisms and offer targets for finding geroprotective interventions.

### Enrichment of PPI on longevity-associated modules

To test the functional coherence of longevity-associated modules, we assessed enrichment in known protein-protein interaction (PPI) networks using STRING (*28*). Applying a stringent threshold (*P* < 1 × 10^−10^) (*26*), we found that 31 of 63 aging/*daf-16* submodules were significantly enriched in proteins known to physically interacted (table S3). Among the most enriched were M27 (*P* = 0; enrichment = 2310), M11 (*P* = 0; enrichment = 9.41), and M2.3 (*P* = 1.17 × 10^−136^; enrichment = 13.7). M27 consisted mostly of ribosomal proteins, while M11 encompassed components of multiple mitochondrial and protein-processing complexes. M2.4 was also enriched for mitochondrial membrane proteins. These results confirm that coexpressed modules are not only transcriptionally coordinated but also functionally integrated at the protein level, reinforcing their biological relevance.

### Preservation of longevity-associated modules in mouse aging organs and their association with frailty

To evaluate cross-species conservation of our longevity-associated modules, we assessed whether *C. elegans* coexpression modules are preserved during aging in mice. Although fewer than 7000 *C. elegans* genes have human or mouse orthologs, 30 modules—including key longevity-associated modules like M27, M2.4, and M11—have at least 50% genes mapped to human orthologs (fig. S6A). Cross-species network preservation analysis confirmed that these longevity-associated modules are conserved across most mouse tissues in the *Tabula Munis Senis* cohort (*29*), except for the liver (fig. S6). We next experimentally investigated whether aging trajectories of these modules were preserved in mouse soleus muscle we collected as a part of our mouse intervention cohorts. We conducted RNA-seq on mouse soleus muscles from four age groups (6, 12, 20, and 28 months) and performed WGCNA (fig. S7A) and module preservation analysis. Consistent with our observations on mouse skeletal muscles in *Tabula Munis Senis* (fig. S6), worm modules M2.4, M11, and M27 were strongly preserved in aging mouse soleus muscle (Zsummary = 12.3 to 25.5; Fig. 5A). Conversely, mouse module M2.1, enriched for mitochondrial genes, intersected with these three preserved worm submodules and showed strong preservation in the worm modules (*Z* = 10.4; Fig. 5, B and C). M2.1 eigengenes followed a similar down-then-up pattern with aging in both sexes like worm M11 and M2.4 (Figs. 5D and 2, C and E) and overlapped with additional worm aging modules (M24, M2.3, and M3.3) (Figs. 5C and 4A). Analysis on mouse module M2.1 also confirmed that it is enriched in essential metabolic genes involved in mitochondria and ribosome (fig. S7B an table S3). These results together suggested that a subset of the longevity-associated modules we identified are preserved and regulated in the similar direction in aging nematodes and in mouse soleus muscles.

**Fig. 5.**
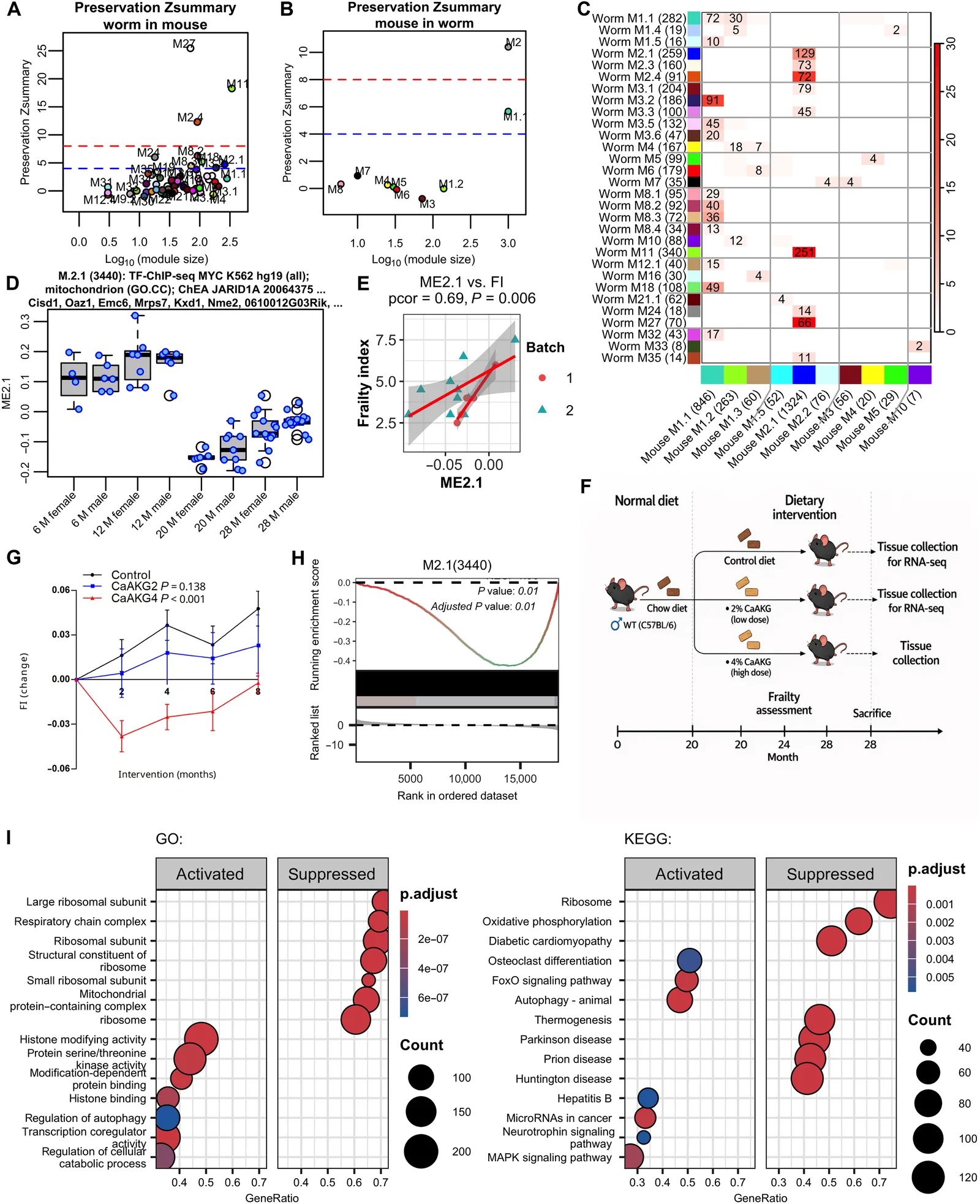
Preservation of longevity-associated modules in mice soleus muscle and module association with health span. (**A**) Preservation Zsummary statistics of mice aging soleus muscle submodules in worm aging/*daf-16* submodules. (**B**) Preservation Zsummary statistics of worm aging/*daf-16* submodules in mice aging soleus muscle submodules. (**C**) Overlapping between the worm aging/*daf-16* sub modules and mouse aging soleus muscle submodules. Only overlaps with *P* values <10^−2^ are shown. (**D**) Expression of mouse M2.1 across ages and sex. (**E**) Association of module eigengene of mouse M2.1 with FI. (**F**) Experimental design for health span assay on CaAKG treatment on mice. Created in BioRender. Huai, W. (2026) https://BioRender.com/hvgdtgh. (**G**) Change in FI upon CaAKG treatment during the period of drug interventions. (**H**) Enrichment of mouse soleus muscles M2.1 in male mice at 28 months upon 8-month treatment of 2% AKG. (**I**) GO (left) and KEGG (right) enrichment analysis of CaAKG treatment.

To assess links between module expression and health span in mice, we measured the frailty index (FI) (*30*) in 28-month-old male mice (table S7). Module M2.1 expression was positively correlated with the FI (partial Pearson *r* = 0.69, *P* = 0.006), indicating that higher expression is associated with worse health (Fig. 5E). Mouse module M1.1, which showed moderate preservation in worms (*Z* = 5.66) and overlapped with worm longevity modules M1.1 and M1.5, was negatively correlated with FI in mice (partial Pearson *r* = −0.72, *P* = 0.003), suggesting a protective role of the module (fig. S7C). Analysis on mouse module M1.1 also showed an enrichment in target genes of *FOXO1*, the human ortholog of *daf-16*, suggesting a conserved role of *daf-16*/*FOXO* pathways for longevity (fig. S7B and table S3).

To test whether module perturbation by interventions is conserved across species, we performed RNA-seq on male mice with alpha-ketoglutarate (AKG), a metabolite known to extend life span (Fig. 5F). Mice received either 2 or 4% of AKG starting at 20 months. While 2% showed modest frailty improvement, 4% significantly reduced frailty by 28 months (Fig. 5G and table S7). In soleus muscle of 2% AKG–treated mice, we observed up-regulation of longevity module M1.1 [normalized enrichment score (NES) = 3.46, FDR = 1 × 10^−10^] and down-regulation of aging module M2.1 (NES = −1.96, FDR = 3.05 × 10^−9^) (Fig. 5H and fig. S7C). Pathway analysis revealed suppression of ribosome and oxidative phosphorylation and induction of autophagy and FOXO signaling—pathways known to support longevity and are enriched in our longevity-associated modules (Fig. 5I).

Together, these results demonstrate that several *C. elegans* longevity–associated modules were preserved in mouse soleus muscle and exhibited similar regulatory patterns and associations with health span. This supports the notion that early transcriptomic changes in these modules may be adaptive and that geroprotective interventions targeting these pathways in worms may translate to vertebrates.

### Predicting novel longevity drugs through longevity-associated modules

To overcome the limitations of the reversal approach, we tested whether we could use our validated longevity-associated modules as molecular markers for drug discovery. We firstly studied how the conserved longevity-associated modules we identified in *C. elegans* and mice were perturbed by perturbagens in human cell lines. We selected modules M1.1, M2.4, M11, and M27—based on their consistent association with longevity and conservation between nematode and mouse muscle—and M20, which showed strong association with life span extension. For large modules (>150 genes), we used the top 100 genes by module membership to meet input gene size limit of the CMap; smaller modules (M2.4 and M27) were analyzed in full. Gene orthologs were mapped to human, and the resulting sets were queried against CMap 1.0, a database of transcriptomic profiles of cellular responses to interventions, to identify compounds that were likely to perturb these modules.

We found that several known geroprotective drug classes—HDAC inhibitors, calcium channel blockers, nuclear factor κB inhibitors, and dopamine antagonists—up-regulated longevity modules and down-regulated aging modules (Fig. 6A), consistent with prior evidence in model species (*31*–*34*). Adenosine triphosphate (ATP) synthase inhibitors also modulated all five modules, implicating mitochondrial metabolism in aging. ATP synthase is also known target of AKG, a metabolite that extends life span across species (*35*–*37*). mTOR and phosphatidylinositol 3-kinase inhibitors influenced four modules, except M27. In contrast, MDM2 inhibitors and bile acids up-regulated aging modules and down-regulated longevity modules, potentially reflecting proaging activity. MDM2 is a regulator of p53, and its inhibition may promote cellular senescence (*38*). Genetic perturbations with predicted geroprotective effects included loss of function in 40*S* ribosomal subunits, RNA polymerase, lysine acetyltransferases, and Akt signaling, while proaging changes were linked to disruptions in nonhomologous end joining repair, phospholipases, and integrin beta subunits (Fig. 6B).

**Fig. 6.**
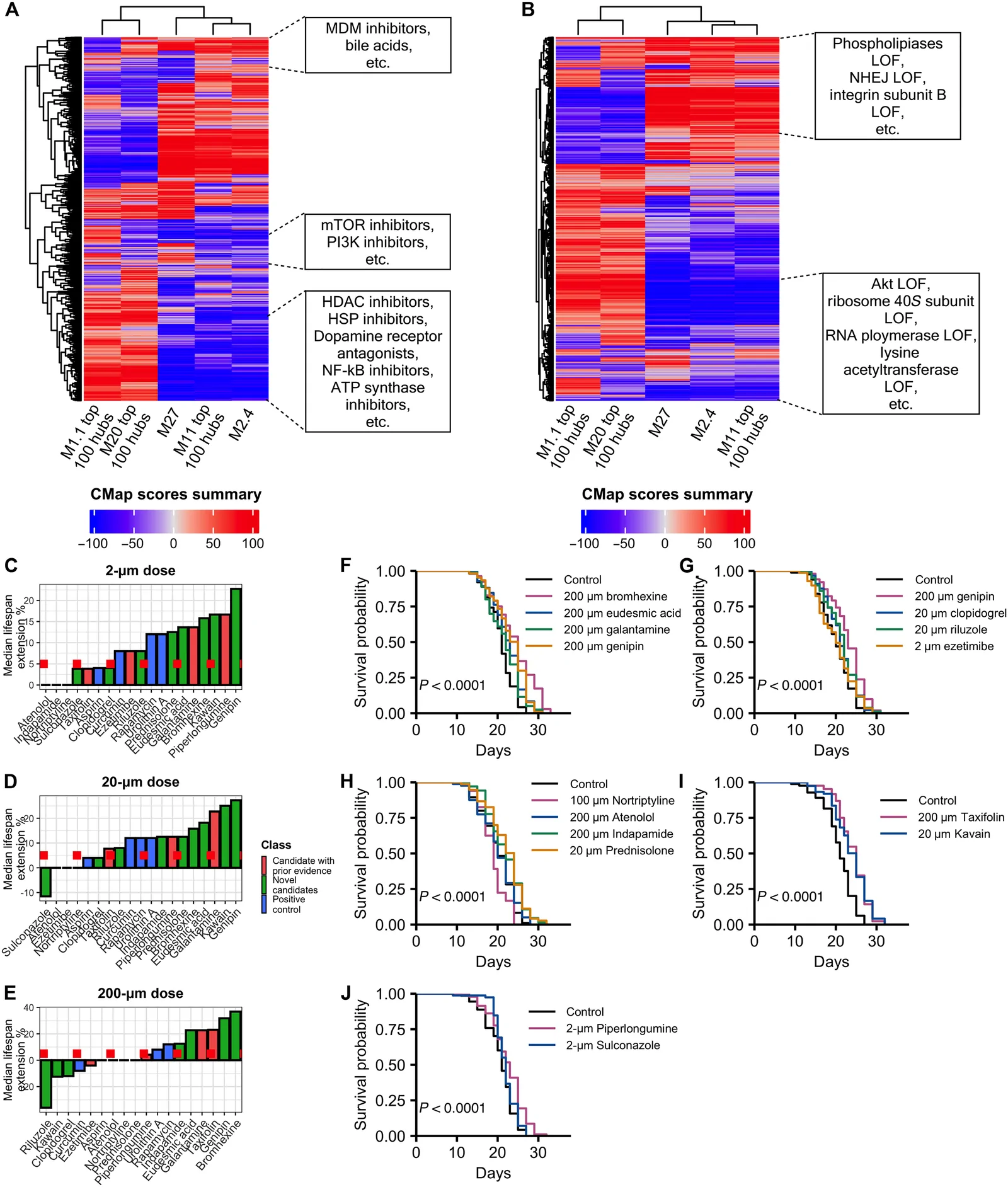
CMap prediction and screening of potential geroprotector using genes from longevity-associated modules. (**A** and **B**) Heatmaps showing the impact of pharmacologic (A) or genetic (B) perturbations on five longevity-associated modules in CMap. (**C** to **E**) Changes of median life span for each candidate drug and positive control relative to solvent controls at doses of 2 μM (B), 20 μM (C), and 200 μM (D) respectively. (**F**) Life span of worms treated with optimized dose of eudesmic acid, galantamine, and genipin, validating results from (C) to (E). (**G**) Life span of worms treated with optimized dose of clopidogrel, riluzole, ezetimibe, and genipin, validating results from (C) to (E). (**H**) Life span of worms treated with optimized dose of atenolol, prednisolone, indapamide, and nortriptyline, validating results from (C) to (E). (**I**) Life span of worms treated with sulconazole and piperlongumine, validating results from (B) to (D). (**J**) Life span of worms treated with kavain and taxifolin, validating results from (C) to (E).

Motivated by these observations, we selected drugs hitting longevity-associated modules as geroprotector candidates for experimental validation. We filtered candidate compounds using three criteria: (i) Food and Drug Administration approval or natural origin; (ii) excluded known geroprotector classes (e.g., mTOR inhibitors); and (iii) not listed in the DrugAge database. Of 223 candidate compounds, we then selected 12 candidates for screening based on how commonly they were used for clinical or traditional medical practice. We additionally selected three with strong selective CMap matches (|score| > 90), yielding 15 total candidates (fig. S8A). Notably, five of these had prior evidence of extending *C. elegans* life span (nortriptyline, galantamine, ezetimibe, piperlongumine, and taxifolin) (*33*, *39*–*42*), supporting our approach. We included four known geroprotectors as positive controls, resulting in a test set of 10 novel candidates, 5 candidates with previous evidence for geroprotective efficacy, and 4 controls (fig. S8B).

To experimentally test the predictive power of our pipelines, we performed a two-step drug screen using three concentrations (2, 20, and 200 μM) under carbenicillin-inactivated bacteria and 5-fluoro-2′-deoxyuridine (FUDR). Candidates extending life span >5% (>1 day) with statistical significance advanced to validation under stricter conditions (cold-inactivated bacteria, no FUDR/carb).

Of the 15 candidates, 11 (73.3%) extended life span by >5% at one or more doses during screening (Fig. 6, C to E), and 9 were effective at multiple doses (fig. S8C). In comparison, three of four positive controls met the same threshold except for aspirin, a control drug that was reported to extend life span in multiple model species, which is potential due to the differences in culturing conditions compared to the literature (*43*–*45*). Nine of our candidates showed stronger median life span extension extent compared to the best-performing positive hits we used (Fig. 6, C to E). One compound previously reported to extend life span in *C. elegans*, nortriptyline, failed in our screen (*33*). In validation, 10 of 11 drugs that showed positive result in the first round were confirmed to have significant life span effects (Fig. 6, F to J), with nortriptyline again showing no benefit—even at the literature-reported 100 μM dose (Fig. 6H).

In total, our screen validated 10 life span–extending compounds in *C. elegans*, 7 of which were entirely novel. In summary, our method achieved a 66.7% hit rate—substantially higher than the 4.4% average from prior unbiased screens (*P* = 0.0014; table S6). These findings show that longevity-associated modules are effective predictors of life span–extending compounds and support their utility in targeted geroprotector discovery.

### Validating impacts of predicted drugs on longevity-associated modules

To experimentally assess whether the life span–extending drugs we found perturb longevity-associated modules as expected, we performed RNA-seq on *C. elegans* treated with two top-performing compounds: bromhexine (a mucolytic drug) and genipin (a natural product from *Gardenia jasminoides*). PCA revealed distinct transcriptomic shifts, with bromhexine-treated worms separating along PC1 and genipin-treated worms along PC3 (Fig. 7A). Differential expression analysis identified 5141 DEGs for bromhexine and 199 for genipin (*P* ≤ 0.01; fig. S9A). Genipin–up-regulated genes were enriched in oxidoreductase and myofibril-related pathways—both linked to extended life span across species (*46*–*48*) (fig. S9, B and C). Bromhexine–up-regulated genes were enriched in metabolic and proteasome pathways, suggesting enhanced proteostasis (fig. S9, D and E).

**Fig. 7.**
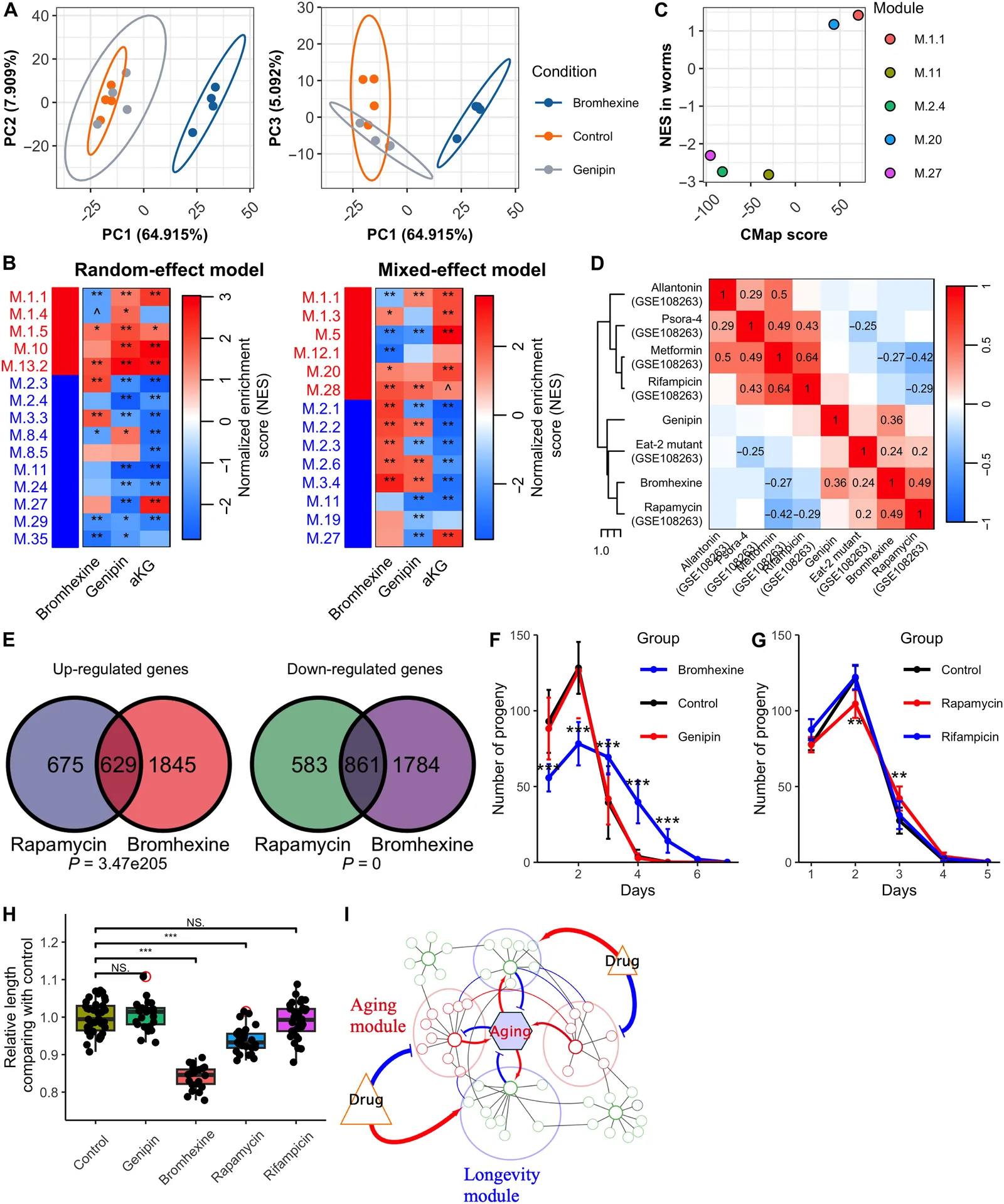
Transcriptomic analysis of bromhexine and genipin and impact of AKG on mouse modules. (**A**) PCA plot of 100 μM bromhexine– and 200 μM genipin–treated worms versus control. (**B**) Heatmap of NES of modules selected as CMap input for bromhexine and genipin. Row color indicates longevity module (red) or aging module (blue). *FDR <= 0.05; **FDR <= 0.01; ***FDR <= 0.001. (**C**) Scatter plot of CMap score and module NES for each input module for genipin treatment. (**D**) Genome-wide Z-statistic associations between drug treatments and DRs across datasets. (**E**) Overlapping of DEG for bromhexine and rapamycin. (**F**) Effects of bromhexine and genipin on reproductive span of *C. elegans*. (**G**) Effects of rapamycin and rifampicin on reproductive span of *C. elegans*. (**H**) Size of the worms treated by rapamycin, rifampicin, genipin, and bromhexine. NS, not significant; *P <= 0.05; **P <= 0.01; ***P <= 0.001. (**I**) Schematics of how drugs slows aging through modulating transcriptomic network.

We next examined whether these drugs targeted predefined aging/*daf-16* modules. Both drugs showed significant positive enrichment in at least two longevity modules and negative enrichment in at least three aging modules (Fig. 7, B and C). Notably, genipin positively regulated all five longevity modules used for CMap input. To explore whether the impact on longevity-associated modules is preserved across species, we additionally compared impact of AKG treatment on modules using our previously performed RNA-seq data. Consistent with the mice result, AKG treatment in worms showed significant positive enrichment in longevity modules and negative enrichment in aging modules (Fig. 7B), suggesting that module perturbation by longevity interventions is conserved across species.

Unexpectedly, bromhexine also activated aging modules M2.1, M2.2, M2.3, and M3.4—a pattern previously observed with rapamycin (Fig. 3B). Their transcriptome-wide Z-statistics were highly correlated (*r* = 0.45), with substantial overlap in up-regulated (629 genes, *P* = 3.47 × 10^−205^) and down-regulated (861 genes, *P* = 0) genes (Fig. 7D), suggesting that these drugs share a unique aging-associated expression program. Phenotyping of drug treatments showed that both rapamycin and bromhexine exhibited extension of reproductive span and reduced body size comparing with genipin and rifampicin (Fig. 7, F to H). These alterations on growth and germline signaling might contribute to the unexpected proaging transcriptomic profiles of bromhexine and rapamycin on many of the longevity-associated modules.

Together, these results confirm that the novel geroprotectors we found modulate longevity-associated modules in *C. elegans* and that factors like growth and reproduction may shape drug-induced aging signatures. These longevity modules represent conserved transcriptomic signatures that can guide geroprotector discovery across species.

## DISCUSSION

We applied machine learning and systems biology approaches to model the aging transcriptome in *C. elegans*, finding that reversal approach was fundamentally limited because a part of transcriptomic changes during aging are adaptive and therefore beneficial for life span. To identify better markers for geroprotector discovery, we built coexpression networks from aging and *daf-16* mutant datasets and identified aging modules (negatively associated with longevity) and longevity modules (positively associated), which better reflect functional aging dynamics. Aging modules may represent essential pathways with trade-offs, while longevity modules reflect genes beneficial for longevity.

Aging modules were enriched for mitochondrial and proteostasis-related genes, consistent with previous studies linking reduced mitochondrial gene expression to extended life span across species (*49*–*52*). In contrast, longevity modules were enriched in genes involved in host defense, muscle maintenance, and *daf-16* targets—which are known prolongevity pathways (*53*–*55*). Expression of many longevity modules like M1.1 increased with age, suggesting that they function as late-acting adaptations.

However, some studies have reported positive correlations between mitochondrial gene expression and life span in long-lived mouse mutants (*56*). We suggest that these associations reflect younger biological ages of long-lived mutants rather than longevity effects as mitochondrial gene expression typically decreases with biological aging. This interpretation is supported by our observation on mouse that geroprotective drugs create an “older” transcriptome early but a “younger” one later, suggesting time-dependent shifts in gene expression.

Mitochondrial modules M2.4 and M11 lost coexpression and reversed expression trends between days 14 and 18 in *C. elegans*—a pattern also seen in mouse soleus muscle at 20 to 28 months. This may reflect a physiological threshold beyond which critical metabolic functions can no longer be down-regulated. We propose that a breakdown in transcriptomic coherence, rather than absolute expression levels, may contribute to late-stage mortality.

We also identified M27, a ribosome-enriched module, as positively associated with age and negatively with life span—a finding consistent with data from African killifish (*57*). Ribosomal proteins are prone to aggregation in aging worms (*58*, *59*), and ribosomal stoichiometry is disrupted in aged mammalian brains (*60*), suggesting that ribosome dysfunction may be a key feature of proteostasis collapse.

While targeting mitochondrial or ribosomal function has extended life span in model organisms through mutations (*61*, *62*), RNA interference (*50*, *58*, *63*), gene deletions (*64*–*66*), or pharmacological interventions (*57*, *67*–*69*), direct inhibition poses risks due to their essential roles. Most known geroprotective drugs—including mTOR inhibitors—directly or indirectly modulate these processes (*70*–*73*). We observed that diverse interventions in *C. elegans* converge on down-regulating mitochondrial and ribosomal modules, suggesting that remodeling energy and protein metabolism—rather than complete suppression—is a shared prolongevity transcriptional state. Therefore, identifying compounds that modulate these pathways at the transcriptomic-module level, rather than directly targeting them at the protein level, may provide a complementary strategy for geroprotector discovery.

Previous studies have consistently reported an age-associated decline in mitochondrial gene expression, implying that mitochondrial gene signatures could serve as biomarkers of aging, and a geroprotective intervention would be expected to reverse this decline over the long term (*29*, *74*–*75*). However, most transcriptomic datasets of perturbagens—such as those from the CMap—captures short-time cellular responses, typically within 96 hours following treatments (*9*, *76*). This introduces a fundamental temporal gap between short-time transcriptomic responses and long-term aging trajectories. Consequently, transcriptomic biomarkers derived from aging or longevity studies in cannot be directly used to query the perturbagen datasets without accounting for this mismatch. This is a part of the reason that most of the studies on predicting geroprotective drugs using transcriptomic data usually ended up with validation on only a few novel drugs (*10*, *11*). Our results highlight that acute transcriptional responses, particularly in mitochondrial and ribosomal pathways, may provide a complementary and practically useful framework for identifying candidate geroprotective interventions from short-term perturbational data.

Building on these insights, we developed a strategy to identify geroprotectors. Rather than reversing aging signatures, we used conserved longevity and aging modules as markers of longevity effect. We hypothesized that effective compounds would down-regulate aging modules and up-regulate longevity modules, shifting the system toward a prolongevity state (Fig. 7I). Of the 15 candidates we tested, 10 extended life span in *C. elegans* under stringent conditions—7 of them novel. Several hits were able to extend life span of *C. elegans* up to 4 days (∼36%) without the presence of FUDR, which is comparable with our previous report on well-characterized life span–extending drugs such as metformin and rapamycin (*17*). Many hits lacked previously known geroprotective mechanisms, highlighting this method’s potential to reveal novel drug classes. However, stress-inducing compounds can also perturb these modules, underscoring the need for careful validation.

Cross-species preservation analysis revealed that worm modules M2.4, M11, and M27—enriched in mitochondrial and ribosomal genes—are conserved in mouse tissues. These overlap with a mouse soleus muscle module, M2.1, which shows a down-then-up expression pattern with age and correlates with frailty. AKG treatment suppressed M2.1 expression, suggesting that its up-regulation at late age is not merely a survivor bias. These findings highlight the importance of tissue-specific gene regulation and offer a framework for developing targeted longevity interventions.

In summary, we developed a modular framework to deconvolute the aging transcriptome and identify transcriptomic signatures associated with longevity. This approach enabled the discovery of novel geroprotective compounds and revealed conserved gene modules that regulate aging across species and tissues.

Our approach has several limitations. First, the meta-analysis relies on aggregating datasets from different laboratories, each potentially using slightly different methodologies. These technical variations may introduce discrepancies in transcriptomic profiles. However, such heterogeneity can also enhance robustness as modules perturbed under diverse conditions are more likely to reflect biologically meaningful signals rather than methodological artifacts. Second, while we identified longevity-associated modules in *C. elegans*, the CMap perturbation profiles are derived from human cancer cell lines. Nevertheless, the fact that the drugs we predicted using the cancer cell line data could extend the life span of *C. elegans* itself is a proof of robustness of our study. Third, interventions that directly target upstream drivers of aging, such as reactive oxygen species, may be more likely to reduce the need for compensatory or adaptive responses and therefore produce a younger biological age signature. Such interventions may be less well captured by our framework. Although we demonstrated partial conservation of these modules in mice, cross-species differences may lead to biases as certain biological pathways may have distinct functional interpretations in worms versus humans.

## MATERIALS AND METHODS

### Experimental design

#### Mouse maintaining

Six- and 12-month-old C57Bl/6 mice (Invivos, Singapore) and 20-month-old C57Bl/6 mice (the Jackson Laboratory, USA) were brought in and allowed to acclimatize 2 months before the start of experiment. Mice are kept in the standard 12/12 light-dark cycle with control feed (Teklad 2918, Envigo, USA) and water ad libitum. The mice were then humanely euthanized, and soleus was immediately placed in RNALater for 24 hours, removed, and immediately frozen until RNA extraction. A separate group of mice were brought in at 17 months old and allowed to acclimatize for 3 months before being randomized into control, low CaAKG (2% CaAKG–supplemented feed), or high CaAKG (4% CaAKG–supplemented feed) group. All feed was provided ad libitum. FI scoring was performed at baseline and every other month. Mice were humanely euthanized at end point, and soleus was immediately placed in RNALater for 24 hours, removed, and immediately frozen until RNA extraction. All experiments followed the National Advisory Committee for Laboratory Animal Research guidelines, and all personnel underwent Responsible Care and Use of Laboratory Animals training before working with mice. The animal experiment is approved by the ethics committee NUS IACUC (ethical approval number: R21-0135).

#### Nematode growing condition

Hermaphrodites *C. elegans* strains wild-type N2, CF1038: *daf-16*(*mu86*) were obtained from Caenorhabditis Genetic Center. Worms were cultured at 20°C on nematode growth medium (NGM) agar plates seeded with OP50-1 strain *Escherichia coli* with streptomycin (200 μg/ml). For compound treatments, all agar plates were prepared from the same batch of NGM agar and treatment plates were supplemented with the respective compounds or vehicles as a control.

#### Microbe strains

A single colony OP50-1 *E. coli* was picked and incubated at 37°C for 20 hours with shaking at 180 rpm in LB broth supplemented with streptomycin (final concentration, 200 μg/ml). Bacterial colony forming units (CFUs) were determined spectrophotometrically at 600-nm wavelength, and the bacterial stock was diluted to 10^10^ CFU with M9 buffers and frozen at −80°C. NGM plates were seeded with bacteria and used for life-span assays without further incubation. Drugs or vehicles were added to a final concentration to the bacteria (in addition to being added to NGM) just before seeding. For the life-span assay for drug screening, bacteria are diluted with LB supplemented with carbenicillin (100 μg/ml), FUDR (50 μM), and streptomycin (200 μg/ml) to 10^10^ CFU and directly seeded to the NGM plates.

#### Mice frailty assessment

To assess the frailty/health span of aged mice, 30 of 31 Clinical Frailty Index measurements outlined by Whitehead *et al.* (*30*) were administered on 28-month mice from their 20^th^ month and every other month. The item scores are 0 (no deficit), 0.5 (mild deficit), and 1 (severe deficit) and included alopecia, loss of fur color, piloerection, coat condition, kyphosis, dermatitis, body condition score, gait, tremor, tumors, distended abdomen, rectal prolapse, vaginal/uterine/penile prolapse, diarrhea, tail stiffening, forelimb grip strength, vestibular disturbance, vision loss, mouse grimace scale, menace reflux, hearing loss, cataracts, corneal opacity, eye discharge/swelling, microphthalmia, nasal discharge, malocclusions, loss of whiskers, breathing rate/depth, and weight. The final FI is the sum of all item scores.

#### *Determination of* C. elegans *lifespan*

Nematodes were age synchronized by bleaching and allowed to hatch, and 120 to 150 young adult worms per condition were transferred to 60-mm culture plates. The life-span experiment was conducted in two conditions: For the initial drug screening stage, the plates are treated with 50 μM FUDR to prevent progeny production, and live OP50-1 was used with supplementation of carbenicillin (100 μg/ml) to prevent bacteria growth. Worms are transferred to fresh plates every 4 to five days to prevent food exhaustion. For the following validation experiments, we used the cold-inactivated bacteria described in the previous section and conducted life-span experiment without the presence of FUDR or carbenicillin. The life-span assay of genipin and bromhexine, two drugs that showed best life-span extension and were subjected to RNA-seq, were performed at least three times.

Worms are transferred to fresh plates every day until progeny production had ceased and every 2 to 3 days afterward. Worms were considered dead when no touch-provoked movement was observed. Animals that crawled off the plate, exhibited extruded internal organs, or displayed an egg-laying defect and bagging were censored. Data assessment was conducted using the “Survival” package in R (*77*) with Kaplan-Meier estimated survival curves compared to each other using the log-rank test.

#### Fertility assay

Age-synchronized (day 3) worms were picked from NGM plate and transferred to fresh 24-well NGM plates containing drug or vehicle. A single worm was used per plate with 10 plates per condition. Worms were transferred to a new wall containing drug or vehicle every day until they ceased laying eggs. Eggs produced by every single worm were incubated at 20°C for 68 hours, and the number of live progeny produced was recorded for each day.

#### Body length measurement

Age-synchronized (day 3) worms were transferred to fresh NGM plates containing drug or vehicle. We took image of worms 48 hours after the treatment using a Leica MZ10F microscope. At least 25 worms per condition were imaged. Images are imported to ImageJ, and body length of nematodes was quantified using the segmented line tool provided by ImageJ. We compared the mean length of worms under different treatments with the length of untreated control worms in each day using *t* test.

#### RNA-seq and preprocessing

Freshly prepared bacteria (OP50-1 *E. coli*) were spotted on 94-cm NGM agar plates on the previous evening and allowed to dry. Synchronized, young adult worms were transferred to fresh plates. For maintaining synchronized populations, FUDR was added to NGM media. Worms are transferred every 2 days into fresh plates to avoid food exhaustion. Worms are harvested at days 6, 8, 11, 14, and 18 and were washed off the plates into 15-ml tubes and then washed several times with fresh buffer until a clear solution was obtained. Clean worm pellets were then frozen and later used for RNA extraction. Total RNA was isolated using Qiagen RNeasy micro kit (Qiagen, Hilden, Germany) following the standard protocol. Afterward, RNA was quantified photometrically with NanoDrop 2000 and stored at −80°C until use.

For the drug treatment, synchronized young adult worms were transferred into plates supplemented with drugs dissolved in dimethyl sulfoxide for 2 days and collected for RNA extraction. For mouse soleus muscles, we collected tissues at different ages and treatments and extracted RNA using Qiagen RNAeasy Mini kit (Qiagen, Hilden, Germany) following the standard protocol.

The integrity of total RNA was measured by Agilent Bioanalyzer 2100. For library preparation, an amount of 2 mg of total RNA per sample was processed using Illumina’s RNA Sample Prep Kit following the manufacturer’s instruction (Illumina, San Diego, CA, USA). Libraries were sequenced using Illumina HiSeq4000 sequencing platform (Illumina, San Diego, CA, USA) in a paired end read approach at a read length of 150 nucleotides. Sequence data were extracted in FastQ format. Sequence data were subjected to quality control FastQC (*78*) and then trimmed using Trimmomatics v0.32 (*79*) to remove adaptors. Processed/cleaned reads were then mapped with STAR (2.7.11b) (*80*) and counted via RSEM 1.3.3 (*81*). The raw count was then imported into R using the R package tximport 1.26.1 (*82*). To filter out low-count genes, we retained only genes with at least 0.5 count per million reads in at least six samples in *C. elegans* to include genes that are likely to be expressed in at least one time point or condition. The remaining genes were then normalized using the variance stabilization method implemented in DESeq2 (*83*).

### Statistical analysis

#### Preprocessing the aging and daf-16 RNA-seq data

We used the network construction methodology described in Esmaili *et al.* (*22*). To filter out outlier samples, we firstly transformed the raw counts with the variance stabilization in DESeq2. Next, we calculated the intersample connectivity using Euclidean intersample distance using the scaled expression profiles of the top 9000 genes with the highest expression and transformed the intersample connectivity into *z* scores.

We removed the outlier in each sample dataset generally using thresholds *z* = −6. For our aging dataset, no sample was categorized into outliers. For the drug combination dataset, two samples were removed as outliers using the same method. To remove potential batch effect within the dataset, we used two different methods: The first one was to use the surrogated variable analysis (SVA) from package sva (*84*) to detect the within-dataset technical differences. The second method is to use the RUVSeq (*85*) and detect unwanted variables using the replicate samples. The sva was performed on variance stabilized data, while RUVSeq was performed directly on raw counts. We next compared the leading surrogated variables (SVs) and the unwanted factors detected from RUVSeq by calculating the Pearson correlations between variables. The leading SVs that are strongly correlated with at least one unwanted variable from RUVSeq are used for correcting the data. For the aging dataset, the data were corrected with the first two SVs using the empirical Bayes-moderated linear regression implemented in EmpiricalBayesLM() function in WGCNA package. For the drug combination dataset, the data were corrected with the first SVs.

#### Construction of PCA-based transcriptomic clock

Typical transcriptomics contains a larger number of features than the number of samples, which makes the typical machine learning method unfeasible. We previously described the construction of aging clocks with lipidomic and clinical parameters using the PCA to scale down the dimension (*15*, *16*). PCA is a dimension down-scaling method based on the singular vector decomposition, which factorizes the transformed data matrix into a new coordinate system of feature matrix with features replaced by the PCs. PCs are linear combination of initial variables and are ranked on the basis of the original information of variance explained. Therefore, the leading PCs can explain most of the variance of the original data matrix.

To split the dataset into train/test data, we firstly randomly selected 80% wild-type day 6 samples and five of six sample from each older age as training data, and the unselected samples are considered as test data. The training of PCA-based transcriptomic clock was carried out on the variance stabilized training data from the previous section before the correction for internal batch effect since the latent batch effect or variance can be detected by PCA itself. The wild type data across different ages were selected and projected into PC coordinates. The elastic net regression model was trained on training data using the top 20 PC coordinates as input and the chronological ages as dependent variables. Model parameters are optimized using the 10-fold validation by grid search over an 11 ratio from 0 to 1 in 0.01 intervals. Biological age is identical to the chronological age in this cohort because each transcriptome sample represents an average value of the cohort of animals. We used the test data, the *daf-16*(*mu86*) mutant in the dataset, the time-coursed sterile control *fer-15*(*bs26ts*), and *fer-15*(*bs26ts*)*;daf-16*(*mu86*) dataset from adult day 2 (GSE122892), and the drug treatment dataset (GSE108263) to test whether the clock can predict the effect of aging, longevity-associated genetic interventions, and drug treatment. For the testing dataset, we first keep the genes that are used in the PCA training sets, and normalized data using variance stabilization method. We next used the singular vectors derived from the training dataset to project each sample’s normalized feature-space coordinates into the same PC coordinate system of the training set.

#### BitAge application

To apply BitAge to our drug treatment dataset, we firstly converted the gene-level counts to counts per million. We next binarized the data using the make_binary() function provided by BitAge (https://github.com/Meyer-DH/AgingClock/). The biological ages of samples were predicted using the test_prediction() function with the BitAge V2 coefficients.

#### Construction of the network

For *C. elegans*, WGCNA was carried out on the variance-stabilized data corrected for the first two SVs in *aging/daf-16* dataset and the variance-stabilized data corrected for the first SVs in drug combination dataset. To improve the robustness of the WGCNA analysis, we used the weighting method slightly modified from biweight midcorrelation, which is firstly described in Lee et al. (*86*). The weighting method is like the biweight midcorrelation, where the Tukey bisquare-like weight λ for each observed gene is calculated as

$$\lambda ={\left(1-u^{2}\right)}^{2}$$

where $u=\text{min}\left[1,\frac{|x-med\left(x\right)|}{9MAD\left(x\right)}\right]$, and MAD is the median absolute deviation of the gene observation. The MAD is further adjusted to meet the conditions where (i) the 10th percentile of the weight is at least 0.1 and the 40th percentile of the weight is at least 0.9 and (ii) the 40% percentile of the weight λ for each individual group is at least 0.9.

We used weighted correlation with individual sample weights determined above and the “signed-hybrid” network in which negatively correlated genes are considered uncorrelated. Scale-free topology on the *aging/daf-16* dataset showed that the resulting networks are approximately scale-free with the power greater than 5. At power of 8, the mean connectivity is approximately 55 and the median connectivity is approximately 30, which are reasonable thresholds. For the drug intervention dataset, at power of 9, the mean connectivity is approximately 50 and the median connectivity is approximately 20, which are reasonable thresholds. Hence, we used the soft power of 8 for constructing *aging/daf-16* network and 9 for constructing drug intervention network.

The topological overlap matrix (TOM) was calculated using the given soft threshold and the weights calculated above. The modules were constructed from the TOM with the following settings: deepSplit = 3, minimum module size of 30, and merge cut height of 0.12. To improve the robustness of our modules, we also removed genes whose eigengene connectivity to their module eigengene was lower than 0.2 from the modules. To improve the stability of our modules, we randomly subsampled approximately two-thirds of samples in each analysis and created 50 sets of WGCNA module labels, which are used as the stability labels in the Dynamic Tree Cut to improve the stability of resulting modules. We used two different minimum stability criteria (0.55 and 0.35) to construct two different sets of modules. The higher criterion results in larger and more robust modules, which are noted as main modules, while the lower criterion produces finer split or those main modules, which are named submodules. Submodules are mainly used for subsequent analysis.

We next calculated the module eigengenes, which is the first singular vector of the standardized module expression matrix. The fuzzy module membership, which describes the similarity of gene expression to the eigengene of the given module, is calculated using the hierarchicalconsensusKME() function implemented in WGCNA package. The top 30 genes with highest module membership within each module are considered as hub genes.

We carried out the same approach on six mouse aging organs (liver, heart, lung, muscle, brain, and small intestine) from the Tabula Muris Senis cohort (https://tabula-muris-senis.sf.czbiohub.org) and the mouse aging soleus muscles data we obtained. The raw counts of mouse aging organs from the Tabula Muris Senis cohort are obtained from the R package TabulaMurisSenisData (*87*). The 1-month samples are excluded from the analysis since mice are potentially in developmental stage at the point. Each tissue was then preprocessed separately. We filtered out low-count genes and preprocessed the data using the same outlier-filtering and batch detection methodologies described above. The WGCNA network construction are performed using the same methodology above with appropriate soft threshold power and merge cut height of 0.15 for each dataset. For all aging organs, the resulting networks are approximately scale-free with the mean connectivity lower than 60 and the median lower than 30. Both the main modules and submodules are obtained using the same minimum stability criteria. For the mouse aging soleus muscle, we chose power of 9, deepSplit = 3, minimum module size of 30, and merge cut height of 0.1 to construct the mouse soleus muscle coexpression network.

#### *Association of modules with ages*, *treatments*, *and mutations*

For the *aging/daf-16* dataset, we used the linear models to relate module eigengenes to individual age contrasts, or mutant contrast with first two SVs as covariates. For mouse soleus muscle dataset, we related module eigengenes to individual contrast of age and sex. We additionally treated ages as a continuous variable and related module eigengenes with it with the first two SVs as covariates. For the drug treatment dataset, we related module eigengenes to individual contrast of drug treatment or mutation versus control, or median life span extracted from the original paper with first SV as covariate.

#### DE analysis

DE analysis was performed using the R package DESeq2 (*83*) version 1.38.3 using default settings. For the design, we used the unwanted variables derived from the RUVseq to correct for the latent batch effect.

#### Association of Z statistics for each dataset

To assess similarity of gene response across different conditions with aging-associated changes, we applied the following strategy: We firstly extracted genes differentially expressed during aging (FDR ≤ 0.05) in our dataset by treating aging as a linear term. We next extracted the *Z* statistics of all aging-associated genes in each drug treatment versus control and computed the correlation of *Z* statistics of these genes between conditions using Spearman correlations. The Spearman correlation matrix were plotted as heatmap with hierarchical clustering using the average-linkage hierarchical clustering. For mouse data, we used GSE131901 to validate our hypothesis. We treated male and female mice separately and obtained DEGs between 12 and 6 months for each sex following the DE analysis described above. Next, we extracted and pooled the *Z* statistics of these genes for each condition versus control and calculated Spearman correlation in males and females separately.

#### Enrichment and annotation of gene sets of DEGs and modules

We used the R package anRichment (https://github.com/plangfelder/anRichment) to calculate the enrichment of WGCNA modules in a large collection of the following gene sets:

1) Gene Ontology (GO);

2) BioSystems gene sets, including KEGG and Reactome;

3) WormEnrichr Anatomic Association from Wormbase 2018;

4) WormEnrichr TF2DNA 2018;

5) WormEnrichr Human Disease from Wormbase 2018;

6) WormEnrichr Gene Interaction Hubs BioGRID 2018;

7) WormEnrichr PPI Hubs BioGRID 2018

Applying the same method, we build a collection of gene sets on mice with the following gene sets: (i) GO, (ii) Biosystems gene sets, 3(iii) ChEA 2022 library, (iv) ENCODE histone modification 2015 library, (v) ENCODE TF CHIP-seq 2015, and (vi) miRTarBase 2017 for annotating mouse modules. Fisher’s exact test was used to evaluate the overlap significance. The genes used in the network analysis are used as background genes for the enrichment analysis. To annotate the WGCNA modules, we combined the top enrichment from each gene sets that have a minimum fraction of the class size of 0.05 to form a merged annotation on each module. Modules with no enrichment with fraction of class size greater than 0.05 were left empty.

For enrichment of DEGs across conditions, we performed Fisher exact test in the collection of gene sets we described. We then kept gene sets enriched in DEG with size between 20 and 500 for visualization.

#### PPI enrichment

The enrichment of protein-protein network was performed using the method previously described (*19*). We firstly downloaded PPI pairs from STRING (version 12.0) database and kept only protein-protein pairs with evidence of binding or forming a physical complex. The network was then filtered to keep proteins in WGCNA analysis. We next performed the hypergeometric testing with the null hypothesis that the frequency of both members of a protein interaction pair presenting inside a module is not greater that the frequency that at least one of the members of the PPI pair does not present in the module.

#### Gene set enrichment analysis

Gene set enrichment analysis (GSEA) are carried out using the R package ClusterProfiler (*88*). DE *Z*-statistics of genes were used as input and ranked with a decreasing order. For gene loading on PCs, we calculated the loadings of each genes on PC1 and then ranked with a decreasing order. GSEA was performed using the modules or gene sets from GO or KEGG as input.

#### Module preservation analysis

Module preservation analysis was performed between the *aging/daf-16* dataset and the drug combination dataset and between the *aging/daf-16* worm datasets and six aging mouse organ datasets, as well as between *aging/daf-16* worm datasets with mouse aging soleus muscles. For module preservation between *aging/daf-16* worm modules and aging mouse organ modules, human ortholog of worm genes were downloaded from Ortholist 2 (OL2) (*89*). The mouse to human sequence homology are obtained from the Mouse Genome Informatics (https://www.informatics.jax.org/downloads/reports/HOM_MouseHumanSequence.rpt) and are merged with the human ortholog to worm genes to obtain the gene ortholog map between *C. elegans* and mice. We then filtered the genes to keep only genes with orthologs in both *C. elegans* and mice by mapping the entrez identifiers between two species using the function mapEntrez() provided by package anRichmentMethods (https://github.com/plangfelder/anRichment) with the ortholog map obtained as the reference. A total of 6680 genes in *C. elegans* were eventually used for measuring module preservation.

We used the modulePreservation function in WGCNA with 500 permutations. The preservation statistics of *aging/daf-16* and drug combination submodules in respect to each other are calculated. The Zsummary score is used to describe the overall preservation statistic of modules. Two thresholds of Zsummary are applied: A Zsummary score greater than 8 is considered as strongly preserved while a Zsummary score greater than 4 is considered as moderately preserved.

#### Module GSVA association with median life span

To calculate the association of module expression and longevity in the drug combination dataset and in public datasets we analyzed, we applied GSVA (*25*) to compute relative module expression changes within datasets. GSVA is an algorithm that calculates an estimated variation of gene set expressions over a sample population. We firstly applied GSVA to compute GSVA scores based on the variance-stabilized data matrix for the coexpression modules we obtained from the *aging/daf-16*(*mu86*) dataset with the kernel function set as “Gaussian.” Next, we obtained the expected median life span for each sample in the original published paper and associate the module GSVA score with expected median life span using the R package limma (*90*) version 3.54.2. Samples with no median life span reported in the original paper or not designed for life span analysis (such as heat shock) are omitted. The SE of log_2_FC obtained from limma was calculated from *t*-statistics using the formula: $\mathrm{SE}=\frac{\text{log}2\text{Foldchange}}{t-\text{statisitcs}}$. The modules with Benjamini-Hochberg (BH)–adjusted *P* values ≤0.05 are considered as significantly associated with expected median life span. For analyzing individual intervention versus control, we associate the module GSVA score with binarized comparison between individual treatment versus control using Limma and obtained log_2_FC for each module as well as the SE.

#### Meta-analysis for conserved longevity-associated modules

To obtain conserved modules associated with life span across datasets, we expanded our data with publicly available RNA-seq datasets from Gene Expression Omnibus (GEO) (*91*), ArrayExpress (*92*), or National Center for Biotechnology Information Sequence Read Archive (*93*) databases. All the public datasets were preprocessed using the same method described in the previous section. The public datasets were selected on the basis of the following criteria: (i) The dataset contains at least eight samples, and (ii) all the samples are performed within 3 days of adulthood are kept for meta-analysis. We also extracted the expected median life span from the original paper. For the datasets with epistasis study, we primarily used the median life span from the epistasis life span analysis. The following datasets meet the criteria after filtering and are used for the meta-analysis: E-MTAB-7272, GSE54853, GSE55988, GSE63075, GSE86493, GSE93724, GSE94077, GSE95240, GSE100482, GSE107134, GSE110984, GSE112981, GSE115531, GSE119485, GSE123531, PRJNA523315, GSE146412, GSE150108, GSE72426, GSE173799, GSE239572, PRJNA508378, and PRJNA479869.

Most of the RNA-seq experiments in the dataset focused on the mechanism of a certain life span–associated pathways, mutations, or interventions. We annotated the dataset with a single term describing the focus of the datasets, such as the main pathways, drugs, or mutant epistasis in the studies. For the dataset PRJNA479869, we split the dataset into three independent epistatic studies based on the epistasis of mutant tested and used the *daf-2* epistasis and *glp-1* epistasis separately for the downstream analysis.

Each dataset was preprocessed using the same procedure described in the previous sections. For identification of longevity-associated modules, we firstly moved unwanted variables and potential outliers for each dataset as described in the previous section and calculated the GSVA scores of each module. We next associated module with expected median life span within each dataset using limma. We obtained the log_2_FC and SE of each module with median life span for each dataset and aggregated the result using the restricted maximum likelihood random effects models using the R package metafor 4.6-0 (*94*). By applying this method, we obtained the aggregated log_2_FC of each module expression with corresponding *P* values, which is further adjusted using the BH adjustment. To improve the robustness of the meta-analysis, we further calculated the leave-one-out *P* value (LOO *P* value), which is defined as the highest *P* values after removal of every dataset one by one, and robust *P* value, which is the lowest *P* value after removal of each dataset one by one. The *P* values of modules significantly associated with life span across datasets are then converted into *z* scores, and a heatmap was generated with hierarchical clustering on modules.

To associating module expression with life span extension in individual conditions, we used a method similar to what was reported in Tyshkovskiy *et al.* (*14*). We firstly collected log_2_ fold change and SE of module GSVA scores for each intervention versus control in each dataset and aggregated 83 interventions with median life span reported. We next applied the mixed-effect model using R package metafor to calculate the *P* value of the association of module expression change with life span effect. We used both mean and SE of log_2_ fold change as input. To remove the batch effect, we considered interventions belonging to the same study and the same control group as a random effect. In addition, we categorized intervention types into three groups: Genetic intervention includes mutation, knock-down, knockout, and overexpression; DR includes *eat-2* mutants or DR; and epistasis includes combination of genetic or drug interventions where the life span extension is compromised comparing with single genetic or drug intervention. The type of interventions was then used as a random effect for the model. We additionally calculated the LOO *P* value and robust *P* value from the meta-analysis. The *P* values calculated are further adjusted using BH adjustment, and modules with strong association with median life span change are obtained on the basis of appropriate threshold on adjusted *P* values.

#### Querying CMap

We selected genes in worm longevity-associated modules M1.1, M2.4, M27, M11, and M20 as input for querying CMap. The human orthologs of *C. elegans* genes in longevity-associated modules are predicted using the Ortholist 2 (*89*) and were applied to query CMap. For modules with size more than 150, we used top 100 genes with highest module membership within the modules as representative genes (hub genes) and converted them into their human orthologs as input for CMap.

The CMap database used the pattern-matching algorithms to compare input gene signatures with gene signatures in diverse perturbagens. CMap generated enrichment scores from +100 to −100 for these perturbagens and ranked depending on their similarities or dissimilarities comparing with the input gene signatures. We expect that a potential antiaging drug would produce negative scores when using aging modules as input and positive scores when using longevity modules as input.

CMap provides perturbation scores on five cell lines. In this study, we used the summary score provided the CMap, which is obtained using the maximum quantile statistics from the vector of normalized connectivity scores of a perturbagen (*76*) as the final score of drug’s perturbation on a module.

#### DrugAge

The DrugAge database (*7*) was accessed in November 2023, and was filtered to include all drugs that significantly increase life span in any model species.

#### GenAge gene selection

The GenAge database was access in November 2023. Since many loss-of-function perturbations of genes result in life span shortening due to collapse of the biological system, we filtered longevity-associated genes so that genes are life span–associated only in the following conditions: (i) Genes whose loss-of-function mutation, RNA interference, or knock-out resulting in life span extension are considered as antilongevity genes; (ii) genes whose overexpression or gain of function resulting in life span extension are considered as prolongevity genes; and (iii) genes whose gain of function result in life span shortening are considered as antilongevity genes.
