# Supplementary material for: Predicting and finding geroprotective compounds through modulating conserved longevity–associated aging modules
Source: Sci Adv. 2026 Sep 30;12(40):eaee3657. doi: 10.1126/sciadv.aee3657 (PMC13626045; doi:10.1126/sciadv.aee3657)
Supplement: Supplementary file 1 [file sciadv.aee3657_sm.pdf]

Science Advances
AAAS

Supplementary Materials for

Predicting and finding geroprotective compounds through modulating conserved longevity–associated aging modules

Weihan Huai *et al.*

Corresponding author: Brian Keith Kennedy, bkennedy@nus.edu.sg; Jan Gruber, bchjg@nus.edu.sg

Sci. Adv. **12**, eaee3657 (2026)
DOI: 10.1126/sciadv.aee3657

The PDF file includes:

Figs. S1 to S10
Legends for tables S1 to S7

Other Supplementary Material for this manuscript includes the following:

Tables S1 to S7

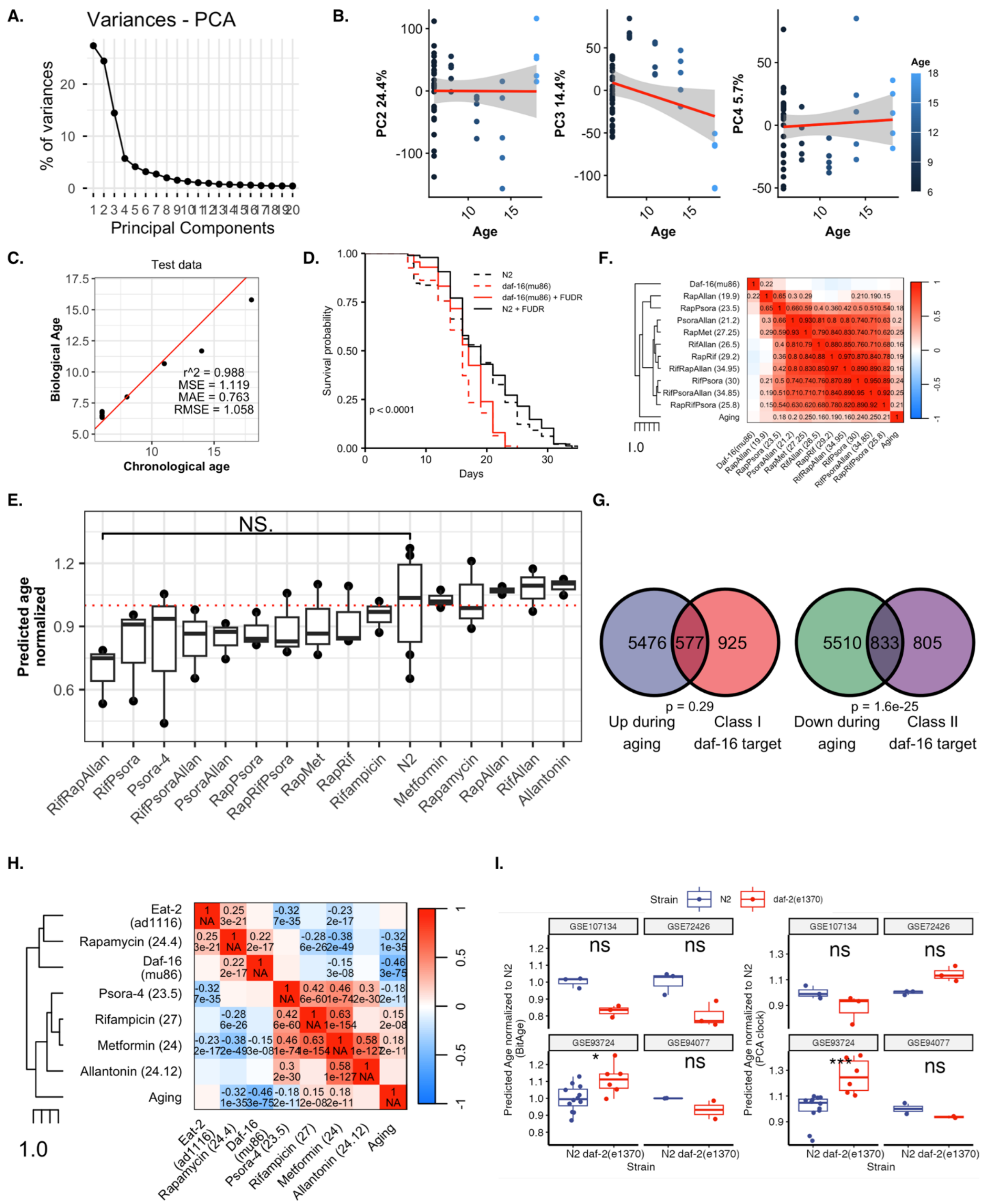

Fig. S1: Details of aging clock and association of treatments with aging. Related to Figure 1.

A. Percentage of variance explained by top 20 PCs.
B. Correlation of PC2, PC3, and PC4 with ages.
C. Prediction of the model on test data left out from our analysis.

D. Lifespan of *daf-16(mu86)* mutants versus wild-type N2 worms.
E. Biological age prediction of drug interventions comparing with control in GSE108263 using BitAge.
F. Correlation of Z statistics of age-associated genes for drug combinations in GSE108263 and this dataset. Only correlations with absolute value >0.15 are shown explicitly. Correlation matrix was clustered using the average hierarchical clustering.
G. (Up) Overlapping top 1000 weighted genes in PC1, genes up-regulated in rifampicin treatment in GSE108263, and genes down-regulated in *daf-16(mu86)* mutants. (Down) enrichment of genes differently regulated during aging and in *daf-16* mutants.
H. Correlation of Z statistics of genes differentially associated with age and *daf-16(mu86)* for individual interventions in GSE108263 and this dataset. Only correlations with absolute value >0.15 are shown explicitly. Correlation matrix was clustered using the average hierarchical clustering.
I. Biological age prediction of *daf-2(e1370)* mutant versus wild-type N2 in four independent datasets, measured by our BitAge clocks and our PCA clock.

A.

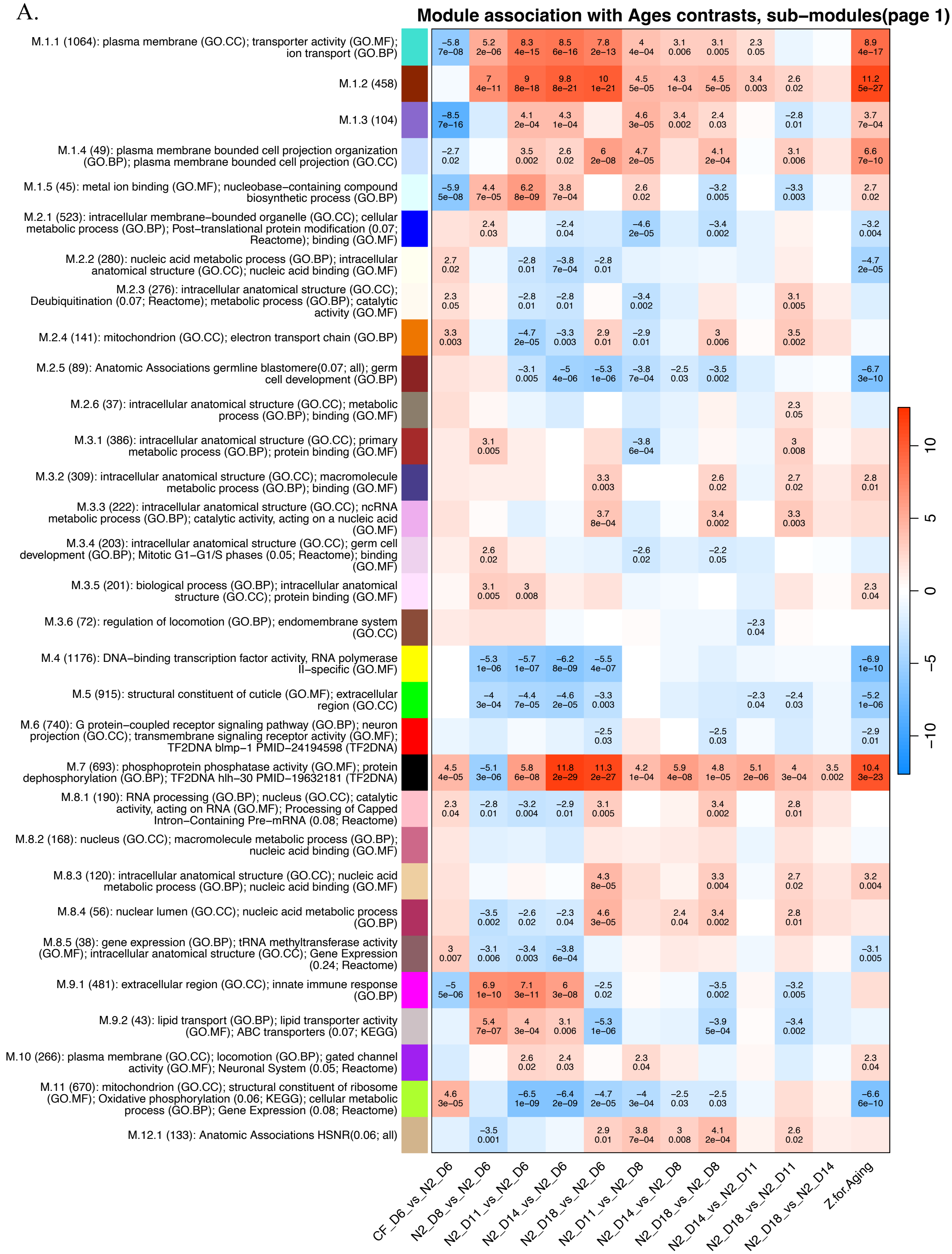
Module association with Ages contrasts, sub−modules(page 1)
M.1.1 (1064): plasma membrane (GO.CC); transporter activity (GO.MF); ion transport (GO.BP)
M.1.2 (458)
M.1.3 (104)
M.1.4 (49): plasma membrane bounded cell projection organization (GO.BP); plasma membrane bounded cell projection (GO.CC)
M.1.5 (45): metal ion binding (GO.MF); nucleobase−containing compound biosynthetic process (GO.BP)
M.2.1 (523): intracellular membrane−bounded organelle (GO.CC); cellular metabolic process (GO.BP); Post−translational protein modification (0.07; Reactome); binding (GO.MF)
M.2.2 (280): nucleic acid metabolic process (GO.BP); intracellular anatomical structure (GO.CC); nucleic acid binding (GO.MF)
M.2.3 (276): intracellular anatomical structure (GO.CC); Deubiquitination (0.07; Reactome); metabolic process (GO.BP); catalytic activity (GO.MF)
M.2.4 (141): mitochondrion (GO.CC); electron transport chain (GO.BP)
M.2.5 (89): Anatomic Associations germline blastomere(0.07; all); germ cell development (GO.BP)
M.2.6 (37): intracellular anatomical structure (GO.CC); metabolic process (GO.BP); binding (GO.MF)
M.3.1 (386): intracellular anatomical structure (GO.CC); primary metabolic process (GO.BP); protein binding (GO.MF)
M.3.2 (309): intracellular anatomical structure (GO.CC); macromolecule metabolic process (GO.BP); binding (GO.MF)
M.3.3 (222): intracellular anatomical structure (GO.CC); ncRNA metabolic process (GO.BP); catalytic activity, acting on a nucleic acid (GO.MF)
M.3.4 (203): intracellular anatomical structure (GO.CC); germ cell development (GO.BP); Mitotic G1−G1/S phases (0.05; Reactome); binding (GO.MF)
M.3.5 (201): biological process (GO.BP); intracellular anatomical structure (GO.CC); protein binding (GO.MF)
M.3.6 (72): regulation of locomotion (GO.BP); endomembrane system (GO.CC)
M.4 (1176): DNA−binding transcription factor activity, RNA polymerase II−specific (GO.MF)
M.5 (915): structural constituent of cuticle (GO.MF); extracellular region (GO.CC)
M.6 (740): G protein−coupled receptor signaling pathway (GO.BP); neuron projection (GO.CC); transmembrane signaling receptor activity (GO.MF); TF2DNA blmp−1 PMID−24194598 (TF2DNA)
M.7 (693): phosphoprotein phosphatase activity (GO.MF); protein dephosphorylation (GO.BP); TF2DNA hlh−30 PMID−19632181 (TF2DNA)
M.8.1 (190): RNA processing (GO.BP); nucleus (GO.CC); catalytic activity, acting on RNA (GO.MF); Processing of Capped Intron−Containing Pre−mRNA (0.08; Reactome)
M.8.2 (168): nucleus (GO.CC); macromolecule metabolic process (GO.BP); nucleic acid binding (GO.MF)
M.8.3 (120): intracellular anatomical structure (GO.CC); nucleic acid metabolic process (GO.BP); nucleic acid binding (GO.MF)
M.8.4 (56): nuclear lumen (GO.CC); nucleic acid metabolic process (GO.BP)
M.8.5 (38): gene expression (GO.BP); tRNA methyltransferase activity (GO.MF); intracellular anatomical structure (GO.CC); Gene Expression (0.24; Reactome)
M.9.1 (481): extracellular region (GO.CC); innate immune response (GO.BP)
M.9.2 (43): lipid transport (GO.BP); lipid transporter activity (GO.MF); ABC transporters (0.07; KEGG)
M.10 (266): plasma membrane (GO.CC); locomotion (GO.BP); gated channel activity (GO.MF); Neuronal System (0.05; Reactome)
M.11 (670): mitochondrion (GO.CC); structural constituent of ribosome (GO.MF); Oxidative phosphorylation (0.06; KEGG); cellular metabolic process (GO.BP); Gene Expression (0.08; Reactome)
M.12.1 (133): Anatomic Associations HSNR(0.06; all)
CF_D6_vs_N2_D6
N2_D8_vs_N2_D6
N2_D11_vs_N2_D6
N2_D14_vs_N2_D6
N2_D18_vs_N2_D6
N2_D11_vs_N2_D8
N2_D14_vs_N2_D8
N2_D18_vs_N2_D8
N2_D14_vs_N2_D11
N2_D18_vs_N2_D11
N2_D18_vs_N2_D14
Z.for.Aging
10
5
0
−5
−10

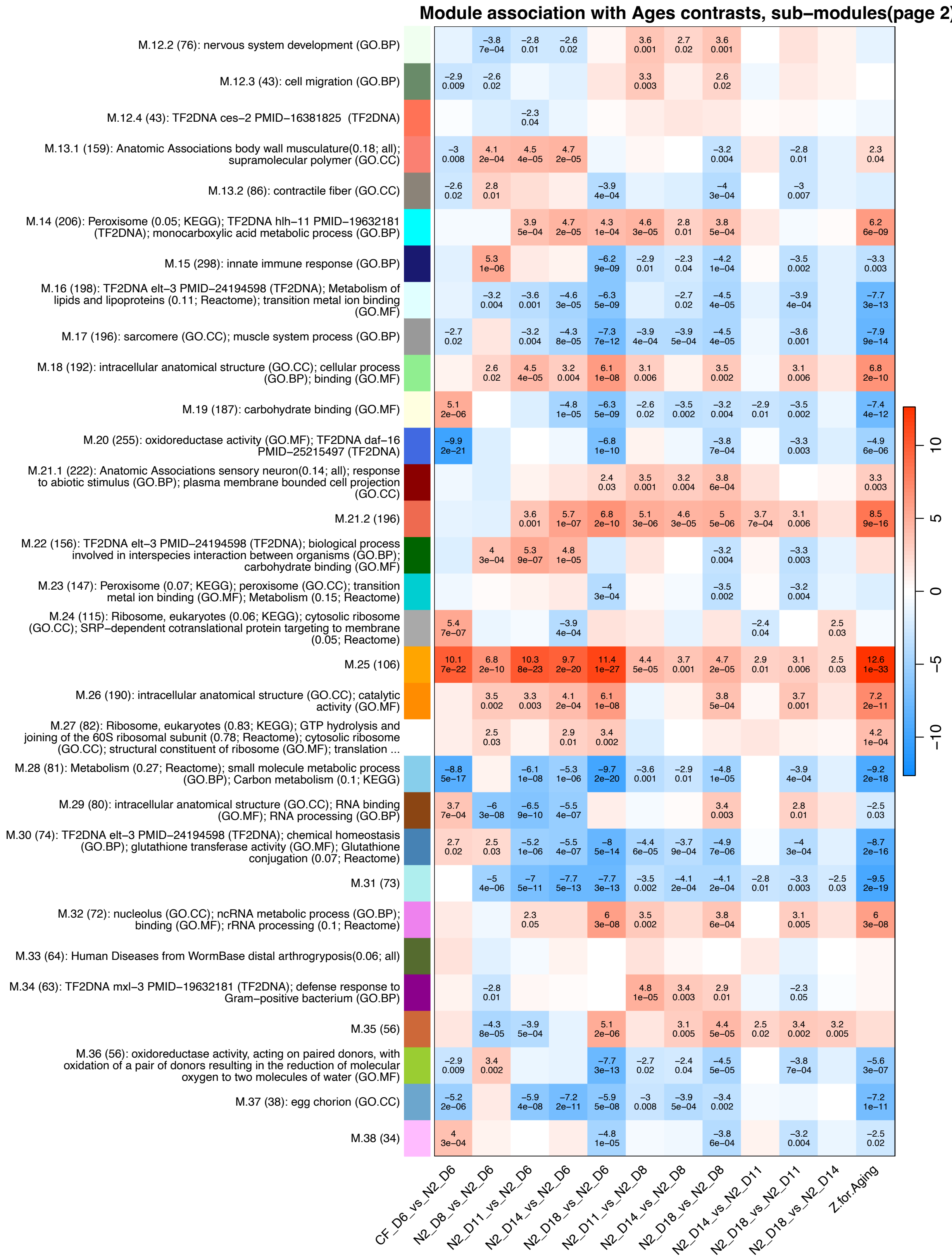
Module association with Ages contrasts, sub−modules(page 2)
M.12.2 (76): nervous system development (GO.BP)
−3.8 7e−04; −2.8 0.01; −2.6 0.02; 3.6 0.001; 2.7 0.02; 3.6 0.001
M.12.3 (43): cell migration (GO.BP)
−2.9 0.009; −2.6 0.02; 3.3 0.003; 2.6 0.02
M.12.4 (43): TF2DNA ces−2 PMID−16381825 (TF2DNA)
−2.3 0.04
M.13.1 (159): Anatomic Associations body wall musculature(0.18; all); supramolecular polymer (GO.CC)
−3 0.008; 4.1 2e−04; 4.5 4e−05; 4.7 2e−05; −3.2 0.004; −2.8 0.01; 2.3 0.04
M.13.2 (86): contractile fiber (GO.CC)
−2.6 0.02; 2.8 0.01; −3.9 4e−04; −4 3e−04; −3 0.007
M.14 (206): Peroxisome (0.05; KEGG); TF2DNA hlh−11 PMID−19632181 (TF2DNA); monocarboxylic acid metabolic process (GO.BP)
3.9 5e−04; 4.7 2e−05; 4.3 1e−04; 4.6 3e−05; 2.8 0.01; 3.8 5e−04; 6.2 6e−09
M.15 (298): innate immune response (GO.BP)
5.3 1e−06; −6.2 9e−09; −2.9 0.01; −2.3 0.04; −4.2 1e−04; −3.5 0.002; −3.3 0.003
M.16 (198): TF2DNA elt−3 PMID−24194598 (TF2DNA); Metabolism of lipids and lipoproteins (0.11; Reactome); transition metal ion binding (GO.MF)
−3.2 0.004; −3.6 0.001; −4.6 3e−05; −6.3 5e−09; −2.7 0.02; −4.5 4e−05; −3.9 4e−04; −7.7 3e−13
M.17 (196): sarcomere (GO.CC); muscle system process (GO.BP)
−2.7 0.02; −3.2 0.004; −4.3 8e−05; −7.3 7e−12; −3.9 4e−04; −3.9 5e−04; −4.5 4e−05; −3.6 0.001; −7.9 9e−14
M.18 (192): intracellular anatomical structure (GO.CC); cellular process (GO.BP); binding (GO.MF)
2.6 0.02; 4.5 4e−05; 3.2 0.004; 6.1 1e−08; 3.1 0.006; 3.5 0.002; 3.1 0.006; 6.8 2e−10
M.19 (187): carbohydrate binding (GO.MF)
5.1 2e−06; −4.8 1e−05; −6.3 5e−09; −2.6 0.02; −3.5 0.002; −3.2 0.004; −2.9 0.01; −3.5 0.002; −7.4 4e−12
M.20 (255): oxidoreductase activity (GO.MF); TF2DNA daf−16 PMID−25215497 (TF2DNA)
−9.9 2e−21; −6.8 1e−10; −3.8 7e−04; −3.3 0.003; −4.9 6e−06
M.21.1 (222): Anatomic Associations sensory neuron(0.14; all); response to abiotic stimulus (GO.BP); plasma membrane bounded cell projection (GO.CC)
2.4 0.03; 3.5 0.001; 3.2 0.004; 3.8 6e−04; 3.3 0.003
M.21.2 (196)
3.6 0.001; 5.7 1e−07; 6.8 2e−10; 5.1 3e−06; 4.6 3e−05; 5 5e−06; 3.7 7e−04; 3.1 0.006; 8.5 9e−16
M.22 (156): TF2DNA elt−3 PMID−24194598 (TF2DNA); biological process involved in interspecies interaction between organisms (GO.BP); carbohydrate binding (GO.MF)
4 3e−04; 5.3 9e−07; 4.8 1e−05; −3.2 0.004; −3.3 0.003
M.23 (147): Peroxisome (0.07; KEGG); peroxisome (GO.CC); transition metal ion binding (GO.MF); Metabolism (0.15; Reactome)
−4 3e−04; −3.5 0.002; −3.2 0.004
M.24 (115): Ribosome, eukaryotes (0.06; KEGG); cytosolic ribosome (GO.CC); SRP−dependent cotranslational protein targeting to membrane (0.05; Reactome)
5.4 7e−07; −3.9 4e−04; −2.4 0.04; 2.5 0.03
M.25 (106)
10.1 7e−22; 6.8 2e−10; 10.3 8e−23; 9.7 2e−20; 11.4 1e−27; 4.4 5e−05; 3.7 0.001; 4.7 2e−05; 2.9 0.01; 3.1 0.006; 2.5 0.03; 12.6 1e−33
M.26 (190): intracellular anatomical structure (GO.CC); catalytic activity (GO.MF)
3.5 0.002; 3.3 0.003; 4.1 2e−04; 6.1 1e−08; 3.8 5e−04; 3.7 0.001; 7.2 2e−11
M.27 (82): Ribosome, eukaryotes (0.83; KEGG); GTP hydrolysis and joining of the 60S ribosomal subunit (0.78; Reactome); cytosolic ribosome (GO.CC); structural constituent of ribosome (GO.MF); translation ...
2.5 0.03; 2.9 0.01; 3.4 0.002; 4.2 1e−04
M.28 (81): Metabolism (0.27; Reactome); small molecule metabolic process (GO.BP); Carbon metabolism (0.1; KEGG)
−8.8 5e−17; −6.1 1e−08; −5.3 1e−06; −9.7 2e−20; −3.6 0.001; −2.9 0.01; −4.8 1e−05; −3.9 4e−04; −9.2 2e−18
M.29 (80): intracellular anatomical structure (GO.CC); RNA binding (GO.MF); RNA processing (GO.BP)
3.7 7e−04; −6 3e−08; −6.5 9e−10; −5.5 4e−07; 3.4 0.003; 2.8 0.01; −2.5 0.03
M.30 (74): TF2DNA elt−3 PMID−24194598 (TF2DNA); chemical homeostasis (GO.BP); glutathione transferase activity (GO.MF); Glutathione conjugation (0.07; Reactome)
2.7 0.02; 2.5 0.03; −5.2 1e−06; −5.5 4e−07; −8 5e−14; −4.4 6e−05; −3.7 9e−04; −4.9 7e−06; −4 3e−04; −8.7 2e−16
M.31 (73)
−5 4e−06; −7 5e−11; −7.7 5e−13; −7.7 3e−13; −3.5 0.002; −4.1 2e−04; −4.1 2e−04; −2.8 0.01; −3.3 0.003; −2.5 0.03; −9.5 2e−19
M.32 (72): nucleolus (GO.CC); ncRNA metabolic process (GO.BP); binding (GO.MF); rRNA processing (0.1; Reactome)
2.3 0.05; 6 3e−08; 3.5 0.002; 3.8 6e−04; 3.1 0.005; 6 3e−08
M.33 (64): Human Diseases from WormBase distal arthrogryposis(0.06; all)
M.34 (63): TF2DNA mxl−3 PMID−19632181 (TF2DNA); defense response to Gram−positive bacterium (GO.BP)
−2.8 0.01; 4.8 1e−05; 3.4 0.003; 2.9 0.01; −2.3 0.05
M.35 (56)
−4.3 8e−05; −3.9 5e−04; 5.1 2e−06; 3.1 0.005; 4.4 5e−05; 2.5 0.02; 3.4 0.002; 3.2 0.005
M.36 (56): oxidoreductase activity, acting on paired donors, with oxidation of a pair of donors resulting in the reduction of molecular oxygen to two molecules of water (GO.MF)
−2.9 0.009; 3.4 0.002; −7.7 3e−13; −2.7 0.02; −2.4 0.04; −4.5 5e−05; −3.8 7e−04; −5.6 3e−07
M.37 (38): egg chorion (GO.CC)
−5.2 2e−06; −5.9 4e−08; −7.2 2e−11; −5.9 5e−08; −3 0.008; −3.9 5e−04; −3.4 0.002; −7.2 1e−11
M.38 (34)
4 3e−04; −4.8 1e−05; −3.8 6e−04; −3.2 0.004; −2.5 0.02
CF_D6_vs_N2_D6
N2_D8_vs_N2_D6
N2_D11_vs_N2_D6
N2_D14_vs_N2_D6
N2_D18_vs_N2_D6
N2_D11_vs_N2_D8
N2_D14_vs_N2_D8
N2_D18_vs_N2_D8
N2_D14_vs_N2_D11
N2_D18_vs_N2_D11
N2_D18_vs_N2_D14
Z.for.Aging
10
5
0
−5
−10

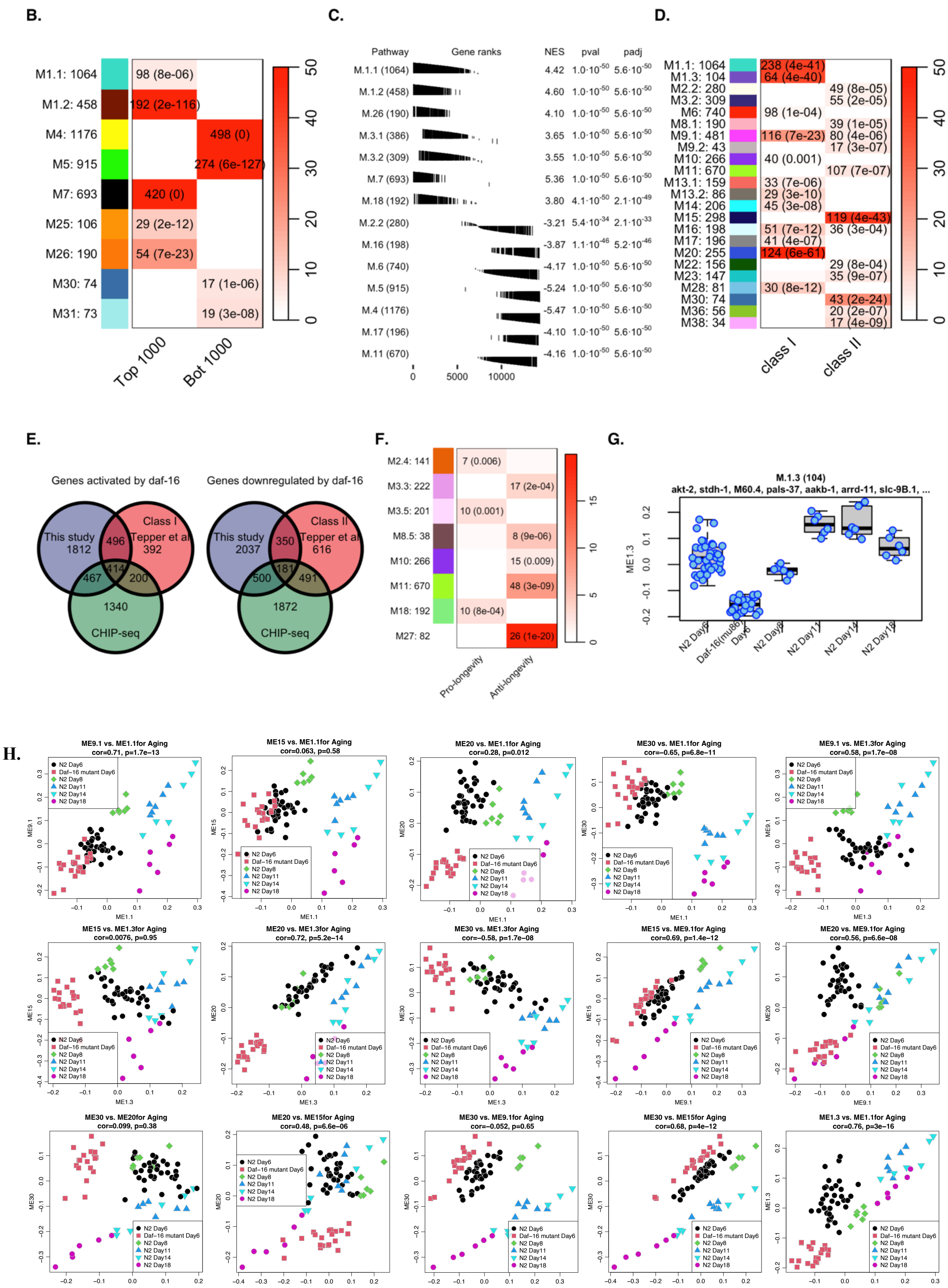
B.
M1.1: 1064
M1.2: 458
M4: 1176
M5: 915
M7: 693
M25: 106
M26: 190
M30: 74
M31: 73
98 (8e-06)
192 (2e-116)
498 (0)
274 (6e-127)
420 (0)
29 (2e-12)
54 (7e-23)
17 (1e-06)
19 (3e-08)
Top 1000
Bot 1000
C.
Pathway
Gene ranks
NES
pval
padj
M.1.1 (1064) 4.42 1.0·10-50 5.6·10-50
M.1.2 (458) 4.60 1.0·10-50 5.6·10-50
M.26 (190) 4.10 1.0·10-50 5.6·10-50
M.3.1 (386) 3.65 1.0·10-50 5.6·10-50
M.3.2 (309) 3.55 1.0·10-50 5.6·10-50
M.7 (693) 5.36 1.0·10-50 5.6·10-50
M.18 (192) 3.80 4.1·10-50 2.1·10-49
M.2.2 (280) -3.21 5.4·10-34 2.1·10-33
M.16 (198) -3.87 1.1·10-46 5.2·10-46
M.6 (740) -4.17 1.0·10-50 5.6·10-50
M.5 (915) -5.24 1.0·10-50 5.6·10-50
M.4 (1176) -5.47 1.0·10-50 5.6·10-50
M.17 (196) -4.10 1.0·10-50 5.6·10-50
M.11 (670) -4.16 1.0·10-50 5.6·10-50
0
5000
10000
D.
M1.1: 1064
M1.3: 104
M2.2: 280
M3.2: 309
M6: 740
M8.1: 190
M9.1: 481
M9.2: 43
M10: 266
M11: 670
M13.1: 159
M13.2: 86
M14: 206
M15: 298
M16: 198
M17: 196
M20: 255
M22: 156
M23: 147
M28: 81
M30: 74
M36: 56
M38: 34
238 (4e-41)
64 (4e-40)
49 (8e-05)
55 (2e-05)
98 (1e-04)
39 (1e-05)
116 (7e-23)
80 (4e-06)
17 (3e-07)
40 (0.001)
107 (7e-07)
33 (7e-06)
29 (3e-10)
45 (3e-08)
119 (4e-43)
51 (7e-12)
36 (3e-04)
41 (4e-07)
124 (6e-61)
29 (8e-04)
35 (9e-07)
30 (8e-12)
43 (2e-24)
20 (2e-07)
17 (4e-09)
class I
class II
E.
Genes activated by daf-16
This study 1812
Class I Tepper et al 392
496
467
414
200
1340
CHIP-seq
Genes downregulated by daf-16
This study 2037
Class II Tepper et al 616
350
500
181
491
1872
CHIP-seq
F.
M2.4: 141
M3.3: 222
M3.5: 201
M8.5: 38
M10: 266
M11: 670
M18: 192
M27: 82
7 (0.006)
17 (2e-04)
10 (0.001)
8 (9e-06)
15 (0.009)
48 (3e-09)
10 (8e-04)
26 (1e-20)
Pro-longevity
Anti-longevity
G.
M.1.3 (104)
akt-2, stdh-1, M60.4, pals-37, aakb-1, arrd-11, slc-9B.1, ...
ME1.3
N2 Day6
Daf-16(mu86) Day6
N2 Day8
N2 Day11
N2 Day14
N2 Day18
H.
ME9.1 vs. ME1.1for Aging cor=0.71, p=1.7e-13
ME15 vs. ME1.1for Aging cor=0.063, p=0.58
ME20 vs. ME1.1for Aging cor=0.28, p=0.012
ME30 vs. ME1.1for Aging cor=-0.65, p=6.8e-11
ME9.1 vs. ME1.3for Aging cor=0.58, p=1.7e-08
ME15 vs. ME1.3for Aging cor=0.0076, p=0.95
ME20 vs. ME1.3for Aging cor=0.72, p=5.2e-14
ME30 vs. ME1.3for Aging cor=-0.58, p=1.7e-08
ME15 vs. ME9.1for Aging cor=0.69, p=1.4e-12
ME20 vs. ME9.1for Aging cor=0.56, p=6.6e-08
ME30 vs. ME20for Aging cor=0.099, p=0.38
ME20 vs. ME15for Aging cor=0.48, p=6.6e-06
ME30 vs. ME9.1for Aging cor=-0.052, p=0.65
ME30 vs. ME15for Aging cor=0.68, p=4e-12
ME1.3 vs. ME1.1for Aging cor=0.76, p=3e-16
N2 Day6
Daf-16 mutant Day6
N2 Day8
N2 Day11
N2 Day14
N2 Day18

Fig. S2: Module association with contrasts of ages, strains, and other external knowledges. Related to Figure 2.

A. Full heatmap of Association of module eigengenes with selected submodules with contrast of ages, mutants, and overall age. The labels of each row indicates the numerical module numbers, the number of genes in the given module, and the top enrichment term for each database.
B. Overlaps of top and bottom 1000 weighted genes from PC1 in PCA clock to aging/*daf-16* modules. Colours represents the hypergeometric enrichment p-values and text represents the size of overlap with respective p-values in the brackets. Only modules with p-value $<10^{-5}$ are shown.
C. Top enrichment of module genes on PC1.
D. Overlaps of *daf-16* downstream targets from Tepper et al., 2013 (19) to aging/*daf-16* modules. *Daf-16* downstream targets are defined as genes associated with FDR <= 0.05 in the original analysis. 'Class I' represents genes that are activated by *daf-16* and 'class II' represents genes repressed by *daf-16*. Only modules with p-value < 0.003 are shown.
E. Venn diagram of DE genes between day 6 *daf-16(mu86)* mutants and day 6 N2, previously identified *daf-16* downstream targets from Tepper et al., 2013 (19), and CHIP-seq results of downstream *daf-16* targets.
F. Overlaps of longevity-associated genes from GenAge (23) to aging/*daf-16* modules. Genes with unannotated or conflicted association with longevity are filtered. Only modules with p-value $<10^{-2}$ are shown.
G. Expression of M1.3 across ages and strains.
H. Scatter plots of module eigengenes between modules with strong enrichment of down-stream *daf-16* targets ($p < 10^{-10}$).

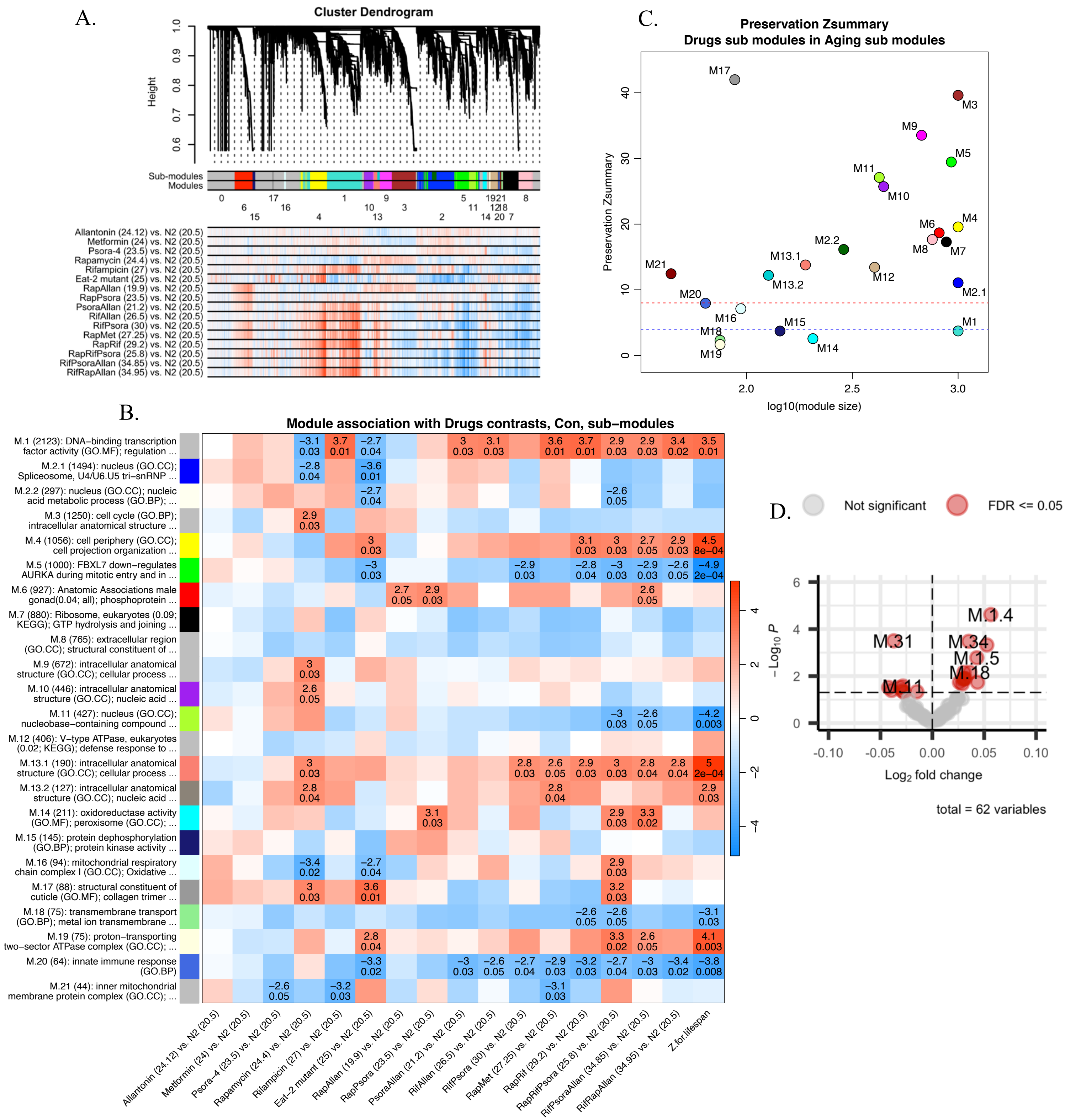

Fig. S3: Construction of drug treatment networks and associating aging/*daf-16* modules with longevities, related to Figure 3.

A. Dendrogram with module colours, labels, and heatmap of individual gene association with each treatment of mutation.

B. Module preservation statistics of drug treatment sub modules on aging/*daf-16* sub modules.
C. Heatmap of module eigengene association with drug treatment compared with control and the expected median lifespan. Volcano plot showing association of GSVA scores of sub-modules with expected median lifespan.
D. Volcano plot showing association of GSVA scores of sub-modules with expected median lifespan.

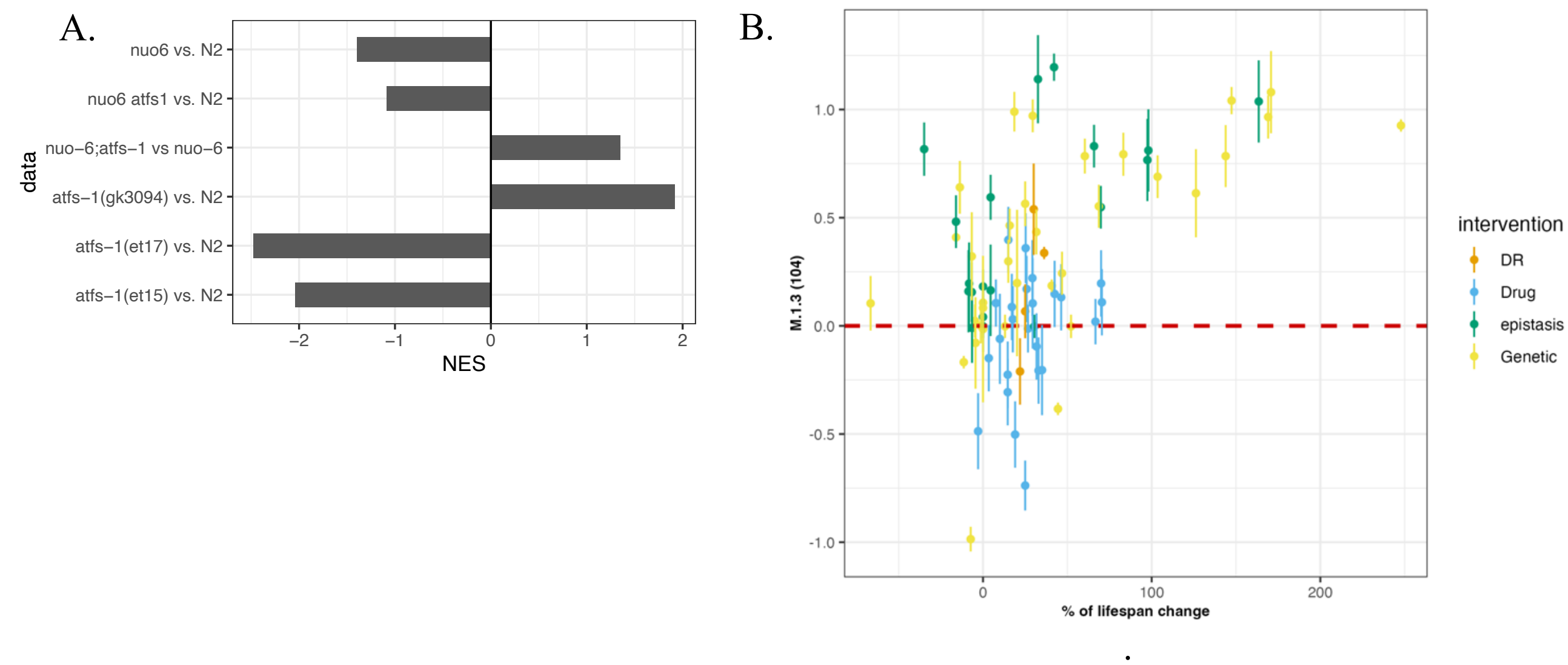

Fig. S4: Meta-analysis to determine longevity-associated modules, related to Figure 4.

- **A.** NES scores of enrichment of module M27 in different interventions in GSE93724.
- B. Association of module M1.3 expression changes with extent of lifespan extension.

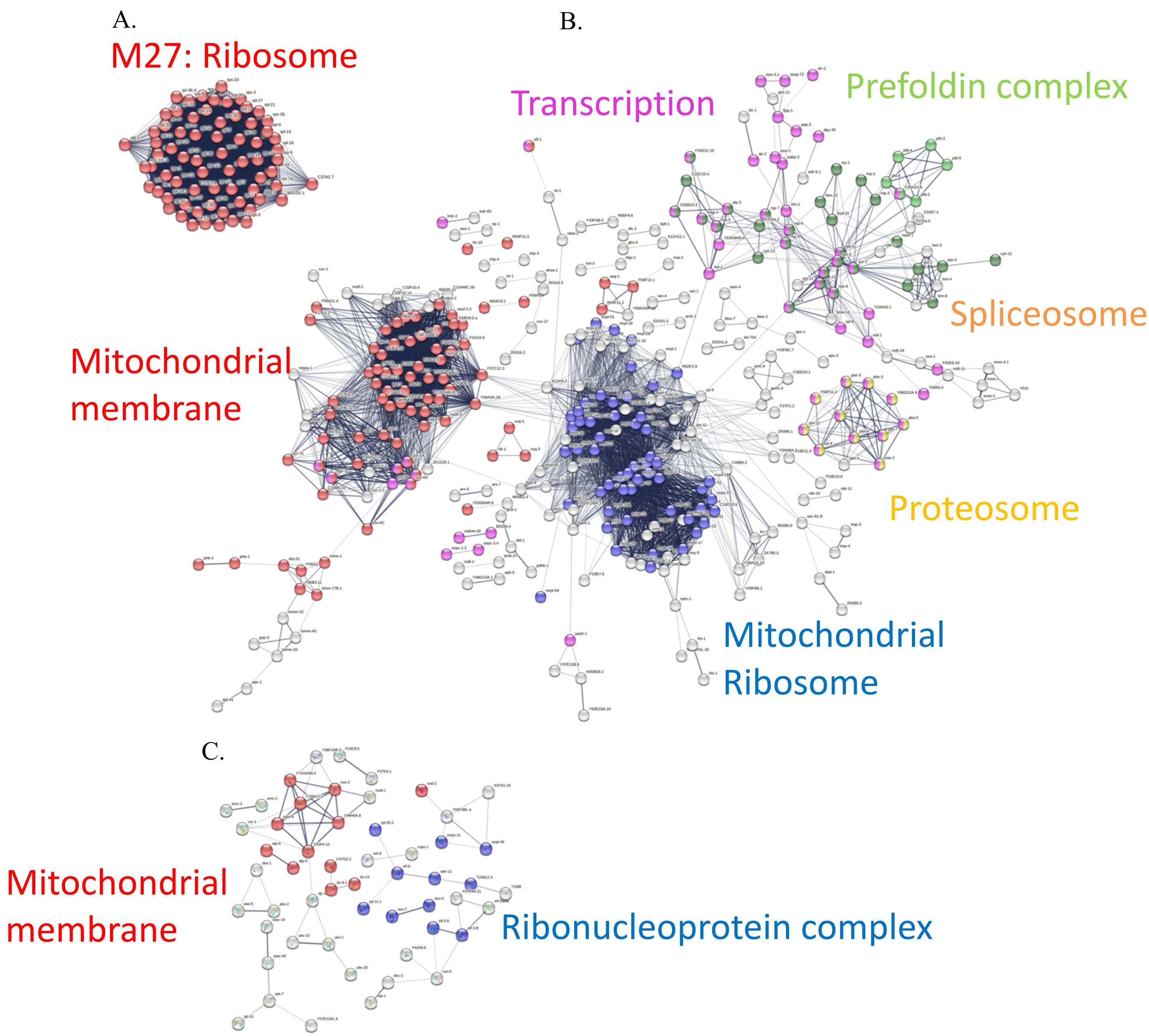

Fig. S5: Protein-protein interaction network of aging modules
A-B. STRING database protein-protein interaction networks of module (A) M27, (B) M11, and (C) M2.4. Functional annotations of several protein complexes are highlight with colors.

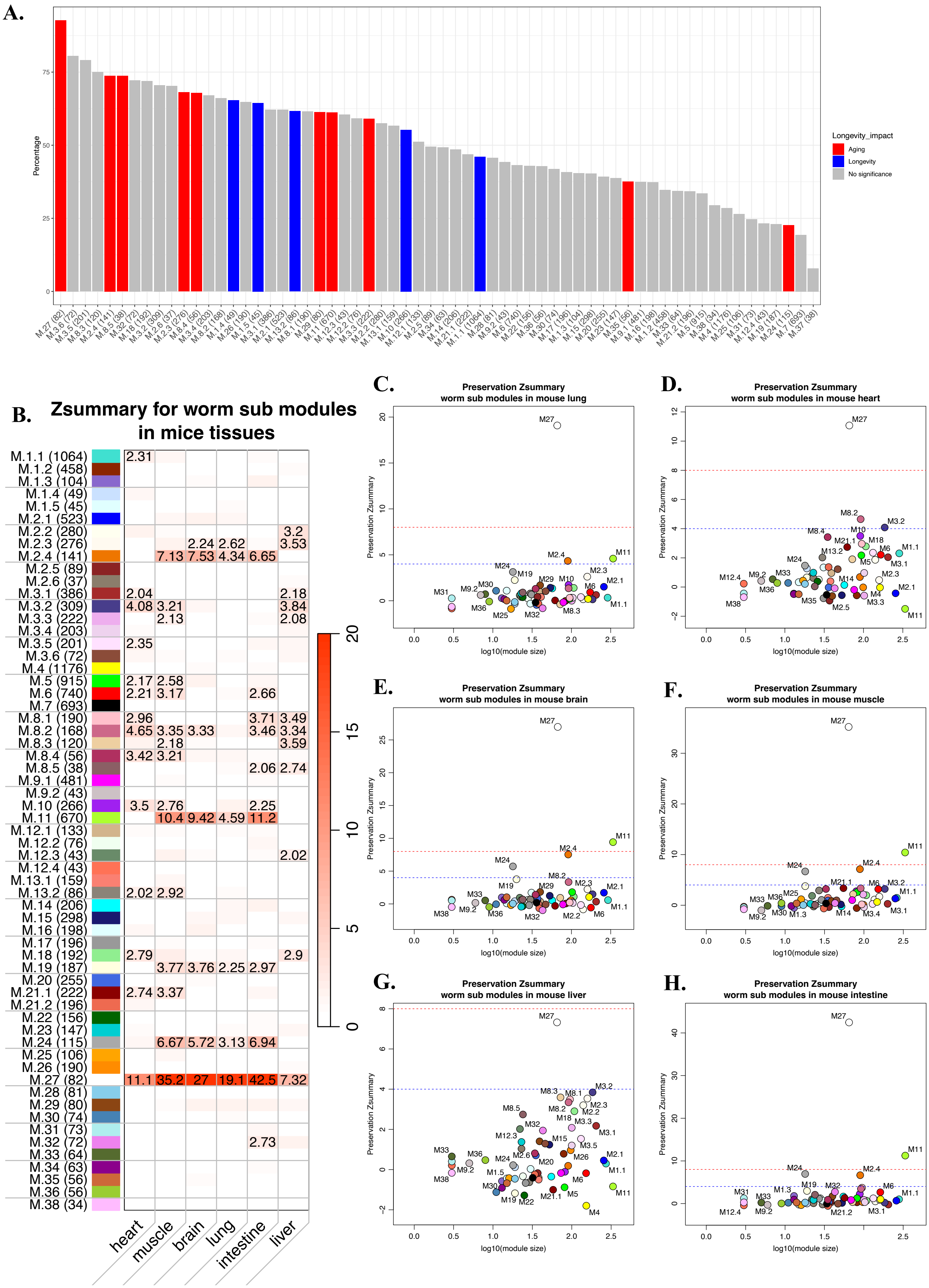

A.
Percentage
Longevity_impact
Aging
Longevity
No significance
B.
Zsummary for worm sub modules in mice tissues
heart
muscle
brain
lung
intestine
liver
C.
Preservation Zsummary worm sub modules in mouse lung
D.
Preservation Zsummary worm sub modules in mouse heart
E.
Preservation Zsummary worm sub modules in mouse brain
F.
Preservation Zsummary worm sub modules in mouse muscle
G.
Preservation Zsummary worm sub modules in mouse liver
H.
Preservation Zsummary worm sub modules in mouse intestine
Preservation Zsummary
log10(module size)

Fig. S6: Preservation Zsummary between aging/*daf-16* worm sub modules and mouse aging organs.

A. Percentage of genes in aging/*daf-16* worm sub modules with human orthologs.

B. Heatmap representation of module preservation Zsummary statistics aging/*daf-16* worms in sub modules or 6 different aging mouse organs. Only Zsummary statistics higher than 2 are labelled.

C-H. Preservation Zsummary statistics of aging/*daf-16* submodules in (C). Mouse lung submodules. (D). Mouse HEART submodules. (E). Mouse muscle submodules. (F). Mouse brain submodules. (G). Mouse liver submodules. (H). Mouse small intestine submodules.

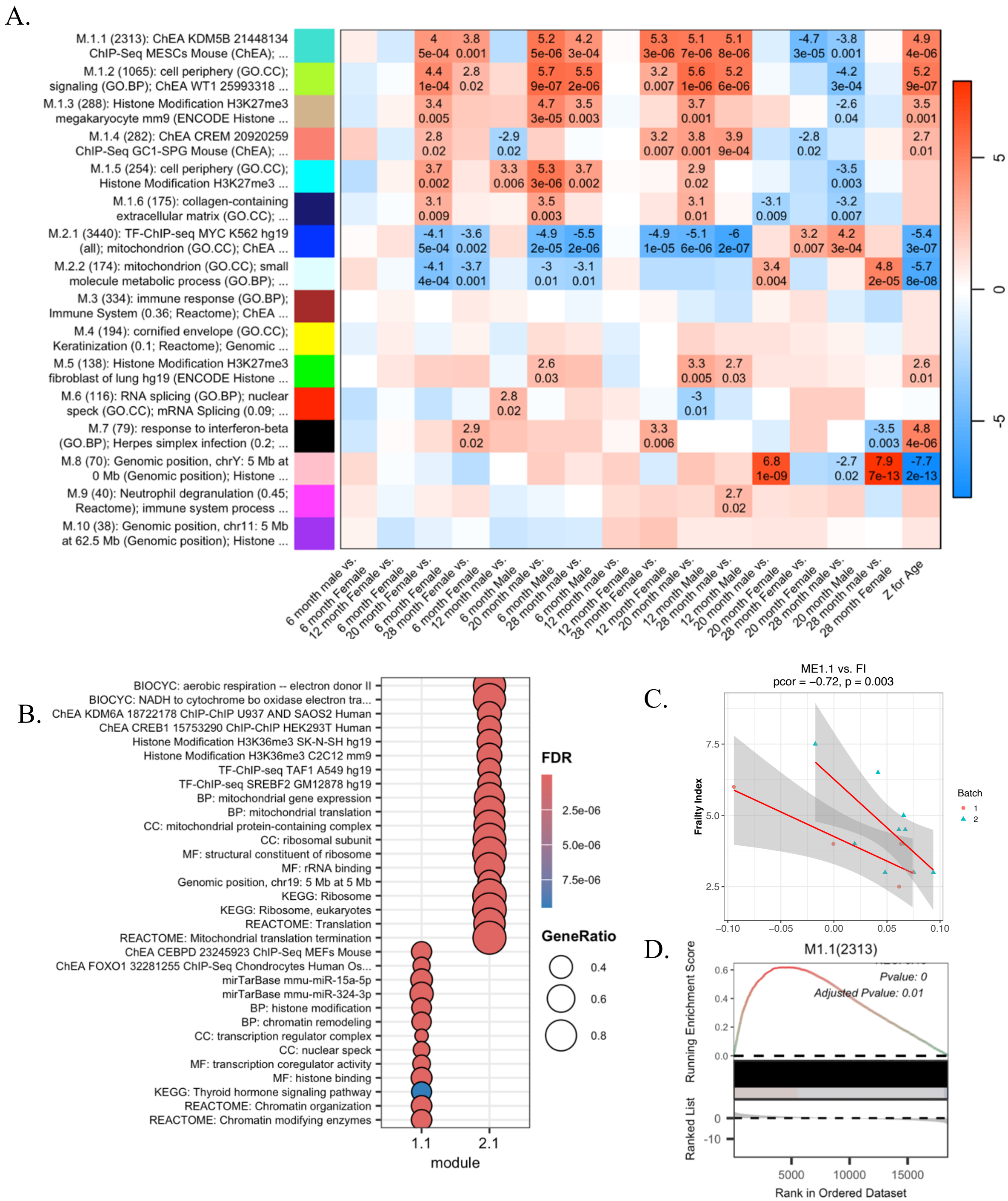

Fig. S7: Association of mouse frailty index with aging soleus muscle modules, related to Fig. 5.

A. Association module eigengenes with submodules with contrast of ages, sex, and overall age. The labels of each row indicates the numerical module numbers, the number of genes in the given module, and the top enrichment term for each database. Modules with no enrichment labels are either having no enrichment term with Benjamini-Hochberg corrected p value < 0.05 or the module-geneset overlapping size is smaller than 10% of the module size. Each column represents a binarized contrast of ages or sex or aging as a

linear variable. The numbers in the heatmap gives the association significance Z statistics with the FDR estimates, which is only shown for those with FDR < 0.05.

B. Dot plot showing significantly enriched terms, ranked by adjusted p-value, for mouse soleus muscle module M1.1 and M2.1. For clarity, only the top two pathways from each gene set category (for example, GO and BP) are shown for each module. The complete enrichment table for all module is provided in Table S3.
C. Association of PC1 with frailty index in 28-month male and female mice.
D. Association of module eigengenes of M1.1 with frailty index in 28-month male mice.

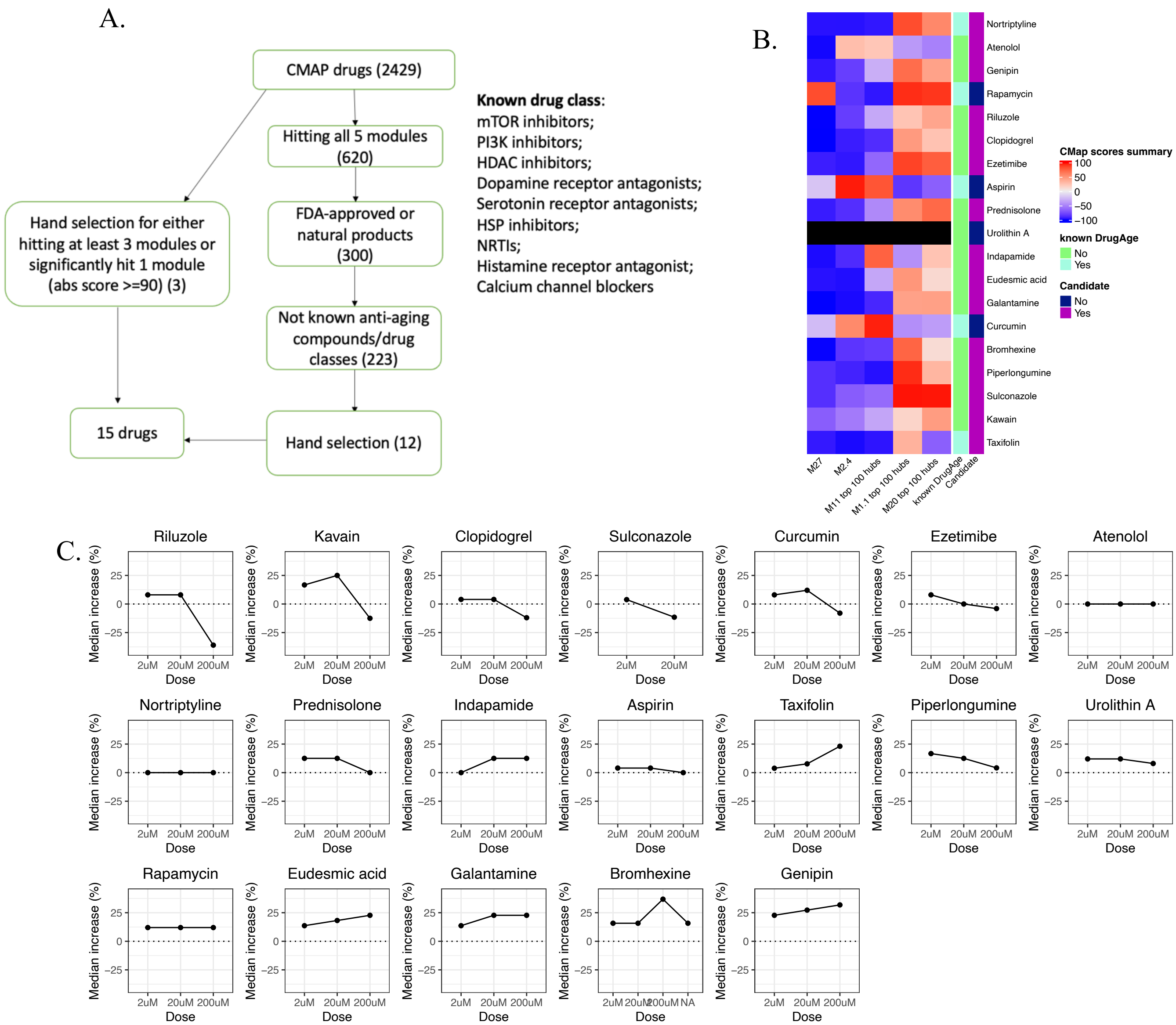

Fig. S8: CMap prediction and screening of potential geroprotector using genes from longevity-associated modules, related to Fig. 6

A. Workflow of drug candidate selection

B. Heatmap of CMap scores of selected candidates and positive controls on each module gene inputs.

C. Dose-response for each drug in the screening. Median lifespan increase are plotted at the y-axis and doses are plotted on x-axis.

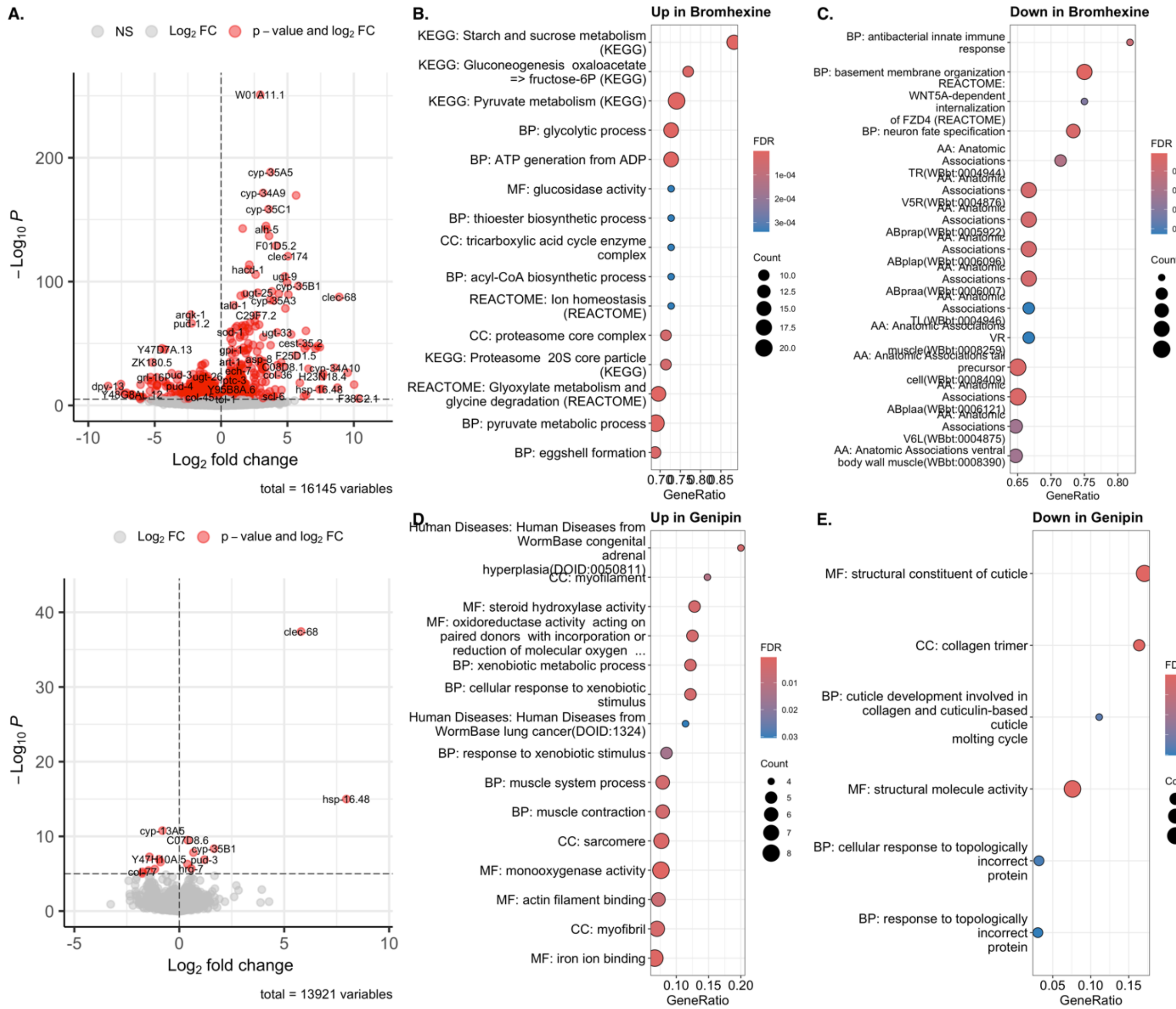

Supplementary Figure 9: Enrichment analysis of DEG by bromhexine & genipin

A. Volcano plot of DEG for bromhexine-treated worms versus control (on the left) and genipin-treated worms versus control (on the right).

B-E. Enrichment of (B) genes up-regulated ($p<0.01$) upon 200uM genipin treatment (C) genes down-regulated upon 200uM genipin treatment (D) genes up-regulated upon 200uM bromhexine treatment and (E) genes down-regulated upon 200uM bromhexine treatment. Only gene sets significantly enriched with enrichment ratio higher than 4 are shown.

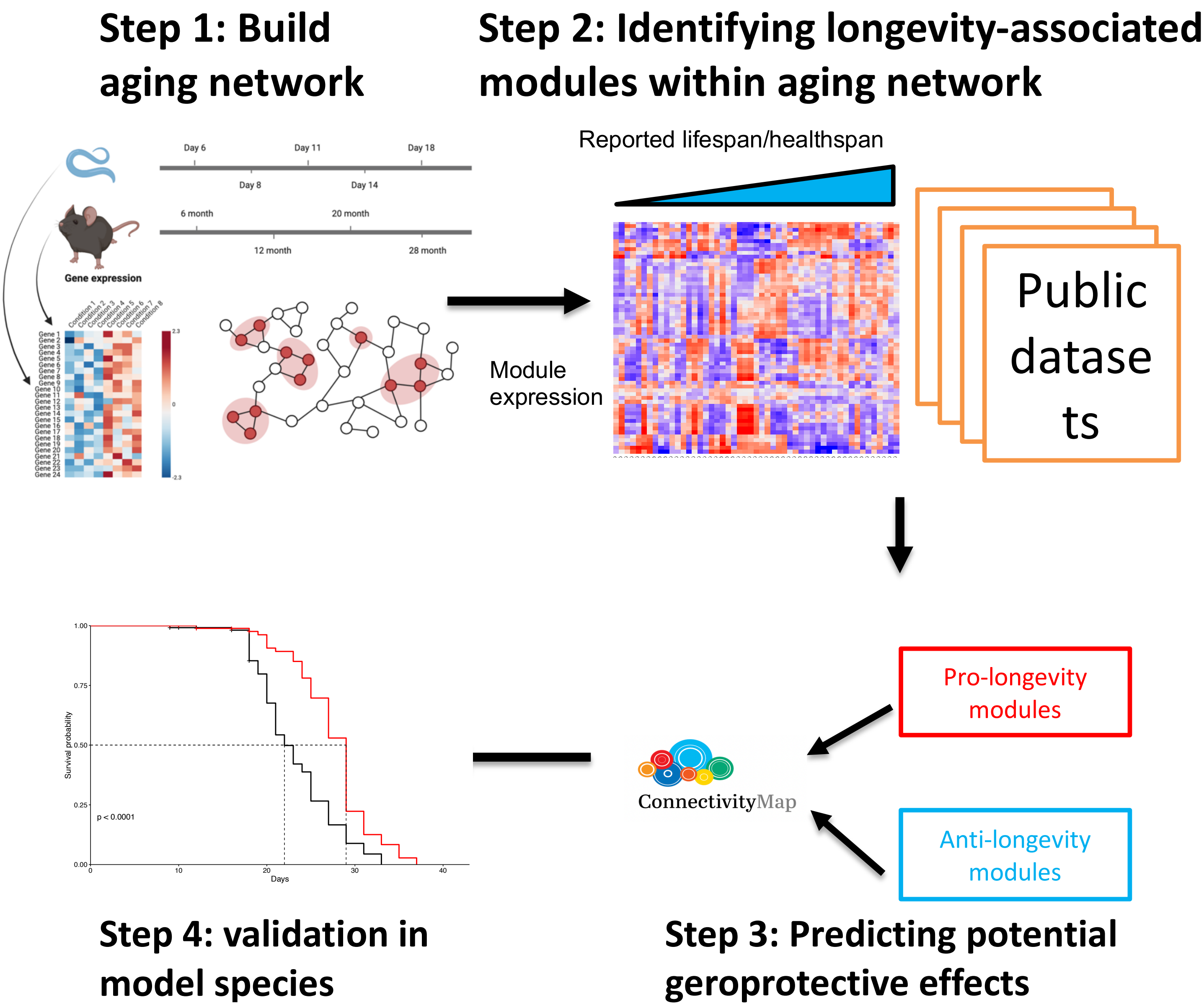

Fig. S10: Illustration of pipeline of identifying novel geroprotectors through genetic-based approach in this study. Top left illustration is created in BioRender. Huai, W. (2026) https://BioRender.com/rk89ggv.

Other Supplementary Materials for this manuscript:

Table S1. Clock prediction of biological age on worms with different dataset, related to Fig. 1.
Table S2. Enrichment of overlapped genes and DEG using anRichment, related to Fig. S1, 7
Table S3. Enrichment of sub modules using anRichment and STRING, related to Fig. 2, S5, and S7
Table S4. Overlaps between DEGs of interventions and modules, related to Fig. 3 & 7
Table S5. Collection and annotation of datasets used for meta analysis and statistics, related to Fig. 4
Table S6. Table S6 Lifespan assay statistics and comparison with unbias screening, related to Fig. 6
Table S7: Frailty index of mice treated with aKG from 20 months to 28 months, related to Fig. 5.
